# Reduced knee extensor torque steadiness and increased motor unit discharge rate variability in young people with patellofemoral pain: a pilot study

**DOI:** 10.1007/s00421-025-06083-8

**Published:** 2025-12-22

**Authors:** Michail Arvanitidis, Deborah Falla, Francesco Negro, Gennaro Boccia, Alberto Rainoldi, Eduardo Martinez-Valdes

**Affiliations:** 1 Centre of Precision Rehabilitation for Spinal Pain (CPR Spine), School of Sport, Exercise and Rehabilitation Sciences, College of Life and Environmental Sciences, University of Birmingham, Birmingham, B15 2TT UK; 2 Department of Clinical and Experimental Sciences, Università Degli Studi Di Brescia, Brescia, Italy; 3 Department of Clinical and Biological Sciences, University of Turin, 10043 Turin, Italy; 4 Neuromuscular Function Research Group, School of Exercise and Sport Science, University of Turin, 10126 Turin, Italy; 5 Department of Medical Sciences, University of Turin, 10126 Turin, Italy; 6 Research Centre for Food and Nutrition (CREA-AN), 00178 Rome, Italy

**Keywords:** Torque steadiness, Patellofemoral knee pain, Motor unit, Vastus medialis, Vastus lateralis

## Abstract

**Purpose:**

The aim of this pilot study was to evaluate differences in vasti muscle motor unit (MU) firing between individuals with current patellofemoral pain (PFP) and asymptomatic controls during submaximal isometric knee extension contractions in both single-joint (knee extension) and multi-joint (leg press) exercises.

**Methods:**

Ten individuals with PFP and ten age- and gender-matched asymptomatic controls performed submaximal isometric contractions (10–70% maximum voluntary isometric contraction; MVIC) during single-joint and multi-joint exercises. High-density surface electromyography assessed MU discharge properties of the vastus medialis (VM) and vastus lateralis (VL), while torque steadiness was quantified using the coefficient of variation of torque.

**Results:**

Neural drive to the vasti muscles was comparable between groups across both exercises however, individuals with PFP exhibited reduced torque steadiness during single-joint compared to multi-joint exercises. This reduction in torque steadiness was accompanied by increased MU discharge rate variability at higher torque levels (50–70% MVIC), particularly for the VL muscle at 70% MVIC. For those with PFP, their pain intensity was also higher during single-joint exercises, which may have further contributed to the aforementioned neuromuscular impairments. Additionally, MU firing-torque relationships revealed neuromuscular adjustments in people with PFP, indicated by significantly lower cross-correlation values during multi-joint exercises compared to the asymptomatic controls.

**Conclusion:**

Physically active people with PFP exhibit reduced torque steadiness, increased discharge rate variability, and potentially altered MU firing-torque relationships during single-joint knee extension exercise. These MU adaptations likely reflect neuromuscular adjustments to ongoing PFP, helping to sustain force production despite impaired motor control and potentially mitigating pain during multi-joint exercises.

**Supplementary Information:**

The online version contains supplementary material available at 10.1007/s00421-025-06083-8.

## Introduction

Patellofemoral pain (PFP) is characterised by pain behind or around the patella (Miao et al. 2015). With an annual prevalence of 23% in adults and 29% in adolescents (Smith et al. 2018), PFP represents a significant clinical challenge. Despite its high prevalence, there is no consensus on its aetiology, largely because the condition is multifactorial (Cook et al. 2012). Proposed risk factors include overuse, direct trauma, anatomical abnormalities, muscle dysfunction, and lower extremity malalignment (Collins et al. 2018; Lankhorst et al. 2012), with neuromechanical factors within the patellofemoral joint playing a key role in the absence of structural injury (Grant et al. 2021).

Research has suggested that altered neuromuscular function of the knee extensor muscles is critical to both the development and maintenance of PFP. The medial and lateral components of the quadriceps generate distinct mediolateral forces at the patella, and thus any imbalance in the activation of the vastus medialis (VM) or vastus lateralis (VL), can disrupt the load distribution across the patellofemoral joint and lead to pathological patellofemoral kinematics (patellar maltracking), which is widely accepted as an underlying mechanism of PFP (Grant et al. 2021). Although many studies have investigated the relative timing of VM and VL activation, a systematic review found only a tendency for the VM to activate later than the VL, with significant unexplained variability across studies (Chester et al. 2008). While measures of the timing of muscle activity offer valuable insights, they only provide a crude indication of the instant when the muscle is active, not the magnitude or quality of neural drive, which depends on both the number and discharge rate of active motor units (MUs). In addition to timing and magnitude of activation, the ability to maintain steady force output during submaximal contractions (torque steadiness), may also influence patellofemoral joint loading (Arvanitidis et al. 2025; Enoka and Farina 2021). Torque steadiness relies on proprioceptive feedback to regulate muscle force output, and this mechanism can be disrupted in the presence of pain (Ager et al. 2020). In individuals with PFP, pain-related interference with proprioceptive input, due to the overlap between proprioceptive and nociceptive neural pathways (Melzack and Wall 1965; Nijs et al. 2012), may impair muscle force control and lead to suboptimal tissue loading with excessive forces applied in unfavourable joint positions (Baker et al. 2002; Pethick et al. 2022). Such alterations can aggravate pain or perpetuate symptoms. Despite its potential relevance in understanding functional deficits and guiding rehabilitation, torque steadiness remains underexplored in people with PFP.

Surface electromyographic (sEMG) amplitude metrics have been previously used to compare muscle activation between individuals with and without PFP, but these measures are only crude indicators of the neural drive to the muscle (Farina et al. 2010), and can be confounded by factors like adipose tissue thickness, normalisation procedures, cross-talk, and MU action potential cancellation (Farina et al. 2004). High-density sEMG (HDsEMG) allows clearer insights through MU decomposition, which overcomes these limitations and provides a more direct assessment of the neural drive to the muscle (Farina et al. 2010). Notably, one study assessing MU behaviour during isometric knee extension in women with PFP found that the firing rate of VL MUs was higher in individuals with PFP compared to asymptomatic controls (Gallina et al. 2018). However, that study was limited to isometric knee extension conditions at very low forces and exclusively in female participants, so it remains unclear whether similar MU behaviour is present in males or during contractions performed at higher force levels or different task demands.

Beyond this previous study, the potential for an exercise to preferentially activate one synergistic muscle over another has long been of clinical interest in the management of PFP. In exercise selection, a distinction is often made between single-joint exercise (also known as open kinetic chain exercise (OKC)) and multi-joint exercises (also known as closed kinetic chain exercises (CKC)). OKC exercises, such as knee extension, typically involve single-joint, non-weight-bearing movements, whereas CKC exercises, such as leg press, are multi-joint and performed under weight-bearing conditions. Although previous conventional sEMG studies have suggested that multi-joint exercises might elicit a more balanced activation between the VM and VL compared to single-joint tasks (Stensdotter et al. 2003), findings have been mixed (Irish et al. 2010; Spairani et al. 2012), leaving no clear consensus on which exercise modality is superior for people with PFP (Witvrouw et al. 2004). Moreover, most previous studies comparing muscle activation across single-joint and multi-joint tasks relied on conventional sEMG, which, as discussed earlier, has inherent limitations. This underscores the need to assess VM and VL MU activation strategies during single-joint exercise and multi-joint exercises in people with PFP, where neuromuscular deficits might be more pronounced, to gain better insights into the neuromuscular effects of these exercises on VM and VL muscles in this population.

This pilot study evaluated differences in vasti muscle MU firing properties between individuals with PFP and asymptomatic controls during submaximal contractions spanning a wide range of target torque levels (from 10 to 70% of maximum voluntary torque) in both single-joint (isometric knee extension) and multi-joint (leg press) exercises, with the aim of comparing neuromuscular control of vasti muscles between these two groups and determining the effects of exercise modality in people with PFP. Multiple contraction intensities were included to assess neuromuscular control across forces typically encountered during daily and sporting activities, as pain and neuromuscular behaviour may vary with different loads (Atkins et al. 2018). Previous work (Gallina et al. 2018) examining MU behaviour in this population, assessed contractions at very low intensities (10% of maximum voluntary isometric contraction; MVIC), providing limited insight into how motor control adapts under higher mechanical demands, where motor deficits and pain may be more evident. Our main hypothesis was that individuals with PFP would show reduced torque steadiness and altered MU discharge behaviour compared with asymptomatic controls. We expected these differences to be more evident at higher contraction intensities and during single-joint contractions, where less support from synergistic muscles requires greater neural drive from the quadriceps and may amplify pain-related adaptations.

## Methods

### Study design and setting

This observational, cross-sectional case–control pilot study with a cross-over design received approval from the Ethical Review Committee at the University of Birmingham, United Kingdom (approval number CM09/03/17–1) and adhered to the Declaration of Helsinki. Data collection spanned from April to July 2017 at a laboratory within the Centre of Precision Rehabilitation for Spinal Pain, University of Birmingham, United Kingdom. The study included two laboratory sessions, scheduled 48 h apart, with participants attending at the same time of day for each session. All participants provided written informed consent before participation. This study is reported according to the STROBE guidelines (von Elm et al. 2008) and the consensus for experimental design in electromyography (CEDE) checklist (Besomi et al. 2024).

Some data from the control group has been previously published (Boccia et al. 2019). The number of controls was reduced from 13 to 10 to ensure age- and gender-matching, with the decision also being informed by retaining only those participants whose signals were of best quality for analysis.

### Participants

Ten physically active individuals with PFP and ten age- and gender-matched asymptomatic controls were recruited from the local Birmingham community, including the University of Birmingham’s student and staff populations, through social media announcements and distributed information leaflets. Participants were eligible for the study if they were men or women aged 18–35 years. The upper age limit of 35 years was chosen to minimise the likelihood of including individuals with degenerative changes, such as osteoarthritis, which become more prevalent with age and could confound the assessment (Shane Anderson and Loeser 2010). Furthermore, all participants were physically active young people, including runners, netball, basketball, and football players, who trained at least three times per week and participated in at least one competitive match or event each weekend. This was not an inclusion criterion, but rather a characteristic of the individuals who were recruited during the study.

The PFP group included participants with anterior knee pain, diagnosed by an experienced physiotherapist (E.M-V., with 12 years of experience in management of musculoskeletal disorders at the time of testing), with emphasis on detailed history-taking (Fulkerson 2002). Diagnosis criteria for PFP included (i) presence of retropatellar or peripatellar pain, (ii) reproduction of retropatellar or peripatellar pain during activities loading the patellofemoral joint (e.g., squatting, stair climbing, prolonged sitting), (iii) positive patellar tilt test, (iv) exclusion of other possible sources of anterior knee pain (e.g., tibiofemoral pathologies, patellar tendinopathy), and (v) exclusion of symptoms referred from the hip or lumbopelvic region (Fredericson and Yoon 2006; Fulkerson 2002; Nunes et al. 2013). To differentiate PFP from other causes of anterior knee pain, the physiotherapist used information from the person’s history, age, and specific physical examination findings. For instance, patellar tendinopathy, unlike PFP, typically presents with pain localised to the inferior pole of the patella or near the tibial tubercle. This pain is often provoked by activities requiring high knee extensor loading, such as jumping or sprinting (e.g., basketball, football), and is accompanied by tenderness on palpation of the patellar tendon, distinct from the diffuse peripatellar pain associated with PFP.

The asymptomatic control group had no history of back or lower limb pain requiring medical attention. Exclusion criteria for both groups included cardiovascular diseases, pregnancy, spinal deformities or surgeries, systemic or inflammatory conditions, rheumatic and neuromuscular disorders, neurological conditions, and lumbar radiculopathy. Participants were also instructed to avoid high-intensity or atypical exercise and refrain from using pain-relief medications 24 h before testing.

### Questionnaires

At the beginning of the session, demographic information was collected for each participant, including age, sex, height, and weight. All participants were also asked to verbally report their level of knee pain after each knee extension contraction at the corresponding target intensity for both the single- and multi-joint exercises, using the 11-point Numerical Pain Rating Scale (NRS), ranging from 0 (no pain) to 10 (worst pain imaginable) (Jensen and McFarland 1993). The NRS is a reliable and valid tool for assessing pain intensity in adults, widely supported in pain measurement literature (Safikhani et al. 2018).

Participants then completed the Kujala Anterior Knee Pain Scale (AKPS) (Kujala et al. 1993), a validated and reliable measure for assessing the severity and functional impact of anterior knee pain (Crossley et al. 2004; Ittenbach et al. 2016; Watson et al. 2005). The Kujala score ranges from 0 to 100, with higher scores indicating better knee function and less pain, while lower scores reflect greater functional impairment and higher pain levels. Additionally, during each submaximal knee extension contraction, participants rated their pain levels using the NRS to quantify the intensity of pain experienced during the task.

### Experimental procedures

The procedures in both experimental sessions were identical, except for the type of exercise performed, either single-joint, or multi-joint, which was assigned in a randomised, balanced order. All measurements were conducted on the dominant limb for the asymptomatic controls and on the painful side for participants with PFP. During each session, the setup allowed participants to view real-time feedback of their exerted torque on a monitor positioned 1.5 m in front of them.

**Fig. 1:**
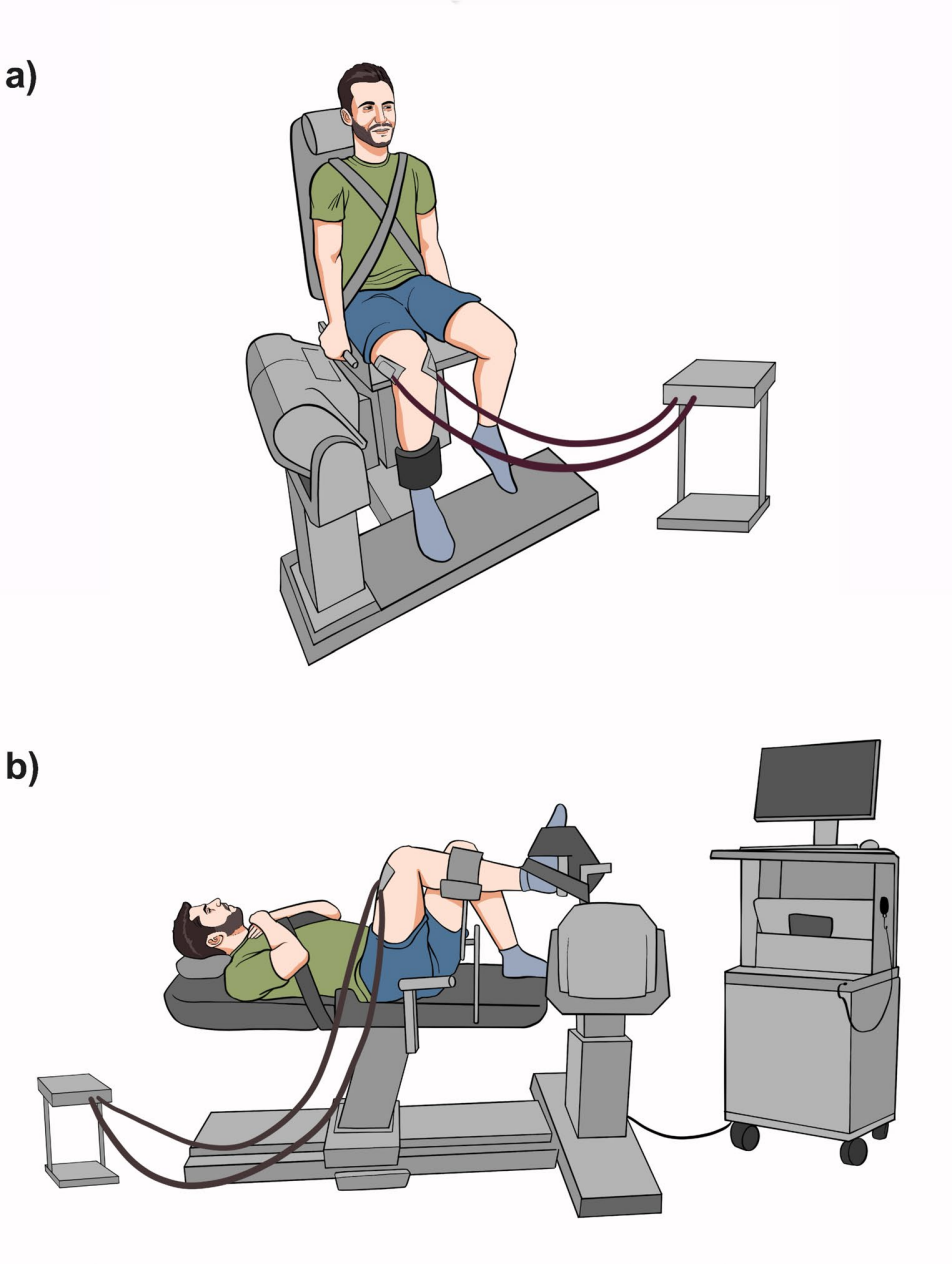
Experimental setup for the single-joint knee-extension exercise (**a**) and the multi-joint leg press exercise (**b**) on an isokinetic dynamometer. In **a**, participants sat upright with their thigh horizontal, while in (**b**) they lay supine with hip, knee, and ankle at 90°. Straps were used to stabilise the pelvis and torso in both exercises. High-density electromyography (HDsEMG) electrodes are also shown, placed over the vastus medialis and vastus lateralis muscles

For the single-joint knee-extension exercise, participants were seated on an isokinetic dynamometer (Biodex System 3, Biodex Medical Systems, Shirley, NY) in an adjustable chair, with the trunk kept vertical and the hip, knee, and ankle joint angles maintained at 90° to position the thigh horizontally. The dynamometer’s rotational axis was aligned with the right lateral femoral epicondyle, and the lower leg was secured to the dynamometer’s lever arm just above the lateral malleolus (Fig. 1a). For the leg press exercise, participants lay supine with their hip, knee, and ankle joints each positioned at a 90° angle, maintaining the tibia in a horizontal position. The rearfoot was fixed to the dynamometer’s lever via a custom-built board, and participants were instructed to push horizontally against the board (Fig. 1b). For both exercises, straps were used to stabilise the pelvis and upper torso, minimising compensatory movements and isolating the knee joint during the contractions.

At the start of each session, participants performed three MVICs, each lasting 5 s, with 2 min of rest between attempts. The highest MVIC value recorded was used as a reference to define submaximal torque levels for that session. Following a 5-min rest period after the MVIC trials, participants performed a series of familiarisation trials at low torque levels. Subsequently, they completed two sets of submaximal isometric knee extension contractions at 10%, 30%, 50%, and 70% of MVIC, in a randomised order. For each participant, the order of contraction intensities was randomised once and then kept consistent across sessions to ensure data comparability and to minimise the potential influence of sequencing-related fatigue that could affect performance in subsequent contractions (Boccia et al. 2019).

Submaximal contractions at 10% and 30% MVIC were held for 30 s, while those at 50% and 70% MVIC were sustained for 15 s. During each trial, participants were instructed to focus on the trapezoidal feedback displayed on the monitor in front of them, follow it as accurately as possible, and maintain a steady force output during the hold phase. The rate of torque increase during the ramp phases was standardised to 10%MVIC/s. Consequently, the ascending and descending ramps took 1 s for 10% MVIC, 3 s for 30% MVIC, 5 s for 50% MVIC, and 7 s for 70% MVIC.

### Data recording

HDsEMG recordings were acquired in a monopolar configuration using two 2D electrode grids with a 13 × 5 arrangement. Each grid contained evenly spaced electrodes (diameter: 1 mm; interelectrode distance: 8 mm; model ELSCH064NM2, OT Bioelettronica, Italy), except for one missing electrode in the upper right corner. Signals were recorded from the VM and VL muscles.

A linear dry electrode array was initially used to determine the optimal position according to muscle fibre orientation. To ensure secure and effective attachment, a double-sided adhesive foam (FOA08MM1305, OT Bioelettronica, Italy) was applied to each electrode. Conductive gel (AC-CREAM, SPES Medica, Genoa, Italy) was then used to fill the electrode cavities, and enhance electrode–skin conductivity. Additionally, participants’ skin was prepared to reduce impedance, which included shaving (if necessary), mild abrasion using an abrasive gel (Nuprep Skin Prep Gel, Weaver and Company, Aurora, Colorado), followed by rinsing with water and drying. Reference electrodes (WhiteSensor WS, Ambu A/S, Ballerup, Denmark) were placed on the participants patella and to an ankle strap equipped with a male clip connector (WS2.1, OT Bioelettronica, Italy). The HDsEMG electrode grids were then placed accordingly, with the electrode columns (13 electrodes) aligned along the muscle fibres, as previously described (Boccia et al. 2019; Laine et al. 2015; Martinez-Valdes et al. 2016).

Torque and EMG signals were sampled at a rate of 2048 Hz and digitised using a 16-bit analog-to-digital converter (Quattrocento, 400-channel EMG amplifier, OT Bioelettronica, Torino, Italy) with a bandwidth of 10–500 Hz at 3 dB. EMG signals were amplified by a factor of 150 and filtered with a bidirectional, 4th-order, zero-lag Butterworth bandpass filter (20–500 Hz). All data were saved to a computer hard drive and processed in MATLAB (version 2023b, The MathWorks Inc., USA). The OTBiolab + software platform was used for data acquisition and visualisation of the torque targets.

### Signal processing

#### Torque signal analysis

Torque signals were low-pass filtered using a fourth-order Butterworth filter with a cutoff frequency of 15 Hz (Contreras-Hernandez et al. 2024; Valli et al. 2024). The highest recorded MVIC (i.e., peak torque) was normalised by dividing the raw torque values (N⋅m) by the individual’s body mass to account for anthropometric differences and facilitate between-group comparisons.

During submaximal contractions, a custom-made MATLAB script was used to plot each participant’s exerted torque, visually identify the steady phase of the contraction (approximately 28 s for the 10% and 30% MVIC contractions and 13 s for the 50% and 70% MVIC contractions), and manually select the start and end points of the time window required for analysis (Arvanitidis et al. 2022). This approach was performed by a researcher blinded to the condition and aimed to exclude the first and last second of the steady part of the contraction, as these periods were when participants typically overestimated or underestimated the requested torque level (Arvanitidis et al. 2024a). The mean and standard deviation (SD) of the signal were then calculated to determine the coefficient of variation of torque (CoV; SD torque / mean torque) (Enoka and Farina 2021).

#### Motor unit analysis

The monopolar HDsEMG signals recorded during all submaximal isometric contractions (10% to 70% MVIC) for both exercises were visually examined using a custom-built MATLAB script. Channels exhibiting excessive noise were identified and removed (< 5% channels were removed). Then all HDsEMG signals were decomposed offline using convolutive blind source separation, a method that has been extensively validated (Negro et al. 2016). Decomposition was performed over the entire duration of the submaximal contractions, and the discharge times of identified MUs were converted into binary spike trains before analysis of discharge characteristics (Del Vecchio et al. 2020).

To ensure accuracy, the decomposition was evaluated using a validated metric (Shilhouette, SIL) that represents the accuracy of the decomposed spike train (Martinez-Valdes et al. 2022), with a threshold set at ≥ 0.90 (Negro et al. 2016). The mean discharge rate and discharge rate variability, expressed as the coefficient of variation of the interspike interval (CoViSi; see details below), were calculated during the stable plateau phase of the torque signal. Recruitment thresholds for each MU were determined as the torque level (%MVIC) at the point when the MU began firing action potentials. Any discharges occurring outside physiologically plausible intervals (separated by ≤ 33.3 ms or ≥ 250 ms, corresponding to 30 and 4 pulses per second, respectively) (Martinez-Valdes et al. 2016) were manually corrected and edited by an experienced researcher using a custom algorithm, following the recent recommendations of Martinez et al. (2023).

#### Cross-correlation coefficient and neuromechanical delay

Neuromechanical interactions between MU rate coding and torque generation were assessed using cross-correlation to evaluate the similarities and delays between fluctuations in MU firing activity and torque. The delay between MU firing activity and torque was used as a measure of neuromechanical delay (NMD) (Del Vecchio et al. 2018). Cross-correlation was performed at two complementary levels to distinguish muscle-specific neural control strategies from global neuromuscular coordination. For the peak cross-correlation (per muscle analysis), MU discharge times were summed separately for each muscle (VM, VL) to generate individual cumulative spike trains (CSTs). These CSTs were then correlated with torque to quantify the relationship between each muscle’s identified MU population and torque output, thereby enabling assessment of muscle-specific neural control strategies. For the cumulative peak cross-correlation analysis, MU discharge times from both muscles were pooled into a single composite CST before correlation with torque (Martinez-Valdes et al. 2022). This approach reflected the integrated neural drive from the entire identified MU pool across the two muscles and provided a global measure of neuromuscular coordination. The cumulative approach fundamentally differs from averaging per-muscle cross-correlation values, as it combines all MU activity before computing the correlation, thereby capturing overall neuromuscular coordination rather than muscle-specific MU firing-torque relationships. These signals, along with torque, were processed by applying a fourth-order zero-phase Butterworth low-pass filter (2 Hz) followed by a high-pass filter (0.75 Hz), as previously described (Martinez-Valdes et al. 2021). The filtered signals were then cross-correlated with torque to determine the similarity in their fluctuations (cross-correlation coefficient) and to compute NMD based on the lag observed in the cross-correlation function (Martinez-Valdes et al. 2022). This analysis was performed for all submaximal contractions (10–70% MVIC) and across both exercises. The cross-correlation coefficient was computed in 5-s segments with 50% overlap (Martinez-Valdes et al. 2022), and the average cross-correlation coefficient and NMD obtained from these segments were reported. This analysis is conceptually similar to correlating the first principal component extracted via principal component analysis, often referred to as the first common component, with torque output, as commonly applied in other studies (Cabral et al. 2025; Negro et al. 2009).

### Statistical analysis

Statistical analyses and graph generation were conducted in R (v4.4.1; R Development Core Team, 2023). MU behaviour was examined using a linear mixed model (LMM) via the *lme4 package* (v1.1.35.5) (Bates et al. 2015), which offers several advantages over conventional analysis of variance (ANOVA). First, it allows the analysis of individual MUs rather than averaging across subjects and conditions, thereby capturing greater data variability. Second, it accounts for the non-independence of observations, a critical factor in repeated-measures designs, as MU discharge data are correlated within individuals. Third, it appropriately models experimental manipulations as fixed effects while accounting for inter-subject variability as a random effect.

For example, the model for the discharge rate variable was specified as:

$${\text{Discharge rate}}\,\sim \,condition \, x \, exercise \, x \, muscle \, x \, torque \, level\, + \,\left( {1 \, | \, participant} \right)$$

This model is interpreted as “the variable of interest (on the left) is predicted by each of the factors on the right”. In this case, the variable of interest (discharge rate) is the dependent variable, while the independent (explanatory) variables, or “fixed effects”, are condition (PFP, control), exercise (single-joint, multi-joint), muscle (VM, VL), and torque level (10%, 30%, 50%, and 70% MVIC). The term (*1 | Participant*) represents the “random effect”, which accounts for variability due to individual differences. This model was applied to all outcome variables except for comparisons of demographic data and questionnaires between groups, for which independent t-tests were used. The model was further adjusted based on the specific outcome; for example, the muscle factor was omitted when analysing the CST-based cross-correlation and torque CoV variables.

After fitting the models, the normality of residuals was assessed using the Shapiro–Wilk test. In cases where normality was violated, residual outliers were removed based on Cook’s distance, using a threshold of four times the standard deviation (Arvanitidis et al. 2024b; Boccia et al. 2019). The statistical significance of fixed effects was determined using Type III Wald F tests with Kenward–Roger degrees of freedom via the ANOVA function from R’s *car package* (v3.1.3). Post-hoc pairwise comparisons were performed using Tukey corrections and least-squares contrasts, as implemented in the *emmeans* package (v1.8.8), and LMM results are reported as mean estimates (M) with 95% confidence intervals (CI). Statistical significance was set at p < 0.05.

The relationships between outcome variables (self-reported measures, HDsEMG, and torque data) were assessed using Pearson’s r via the *Hmisc* package (v5.1.3). Descriptive data, including participant characteristics and data presented in graphs, are reported as means ± standard deviations unless otherwise stated.

## Results

### Participant characteristics

Overall, the age and BMI differences between the groups were not statistically significant, and both groups had the same proportion of men and women. The Kujala score was notably lower in the PFP group, reflecting the presence of patellofemoral pain and suggesting mild-to-moderate self-reported disability. This information is also presented in Table 1.

**Table 1:** Demographic and clinical characteristics of the study participants. Data is presented as mean ± SD

Characteristic	Controls (N = 10)	PFP (N = 10)	P Value
Age (years)	26 (5.6)	23 (5.0)	0.22
Gender (male/female)	6/4	6/4	–
BMI (kg/m^2^)	21.9 (2.5)	23.3 (3.8)	0.34
KUJALA score	100	80.7 (9.1)	–
Isometric knee extension peak torque (N⋅m⋅kg^−1^)	2.81 (0.63)	2.11 (0.46)	0.069
Isometric leg press peak torque (N⋅m⋅kg^−1^)	4.13 (1.10)	3.68 (0.99)	0.069

For all variables except for the correlation analyses, the ANOVA results from the linear mixed models are presented in Supplemental File 1, while the main text highlights both significant and non-significant findings that were considered most meaningful, along with their relevant pairwise comparisons.

### Maximum voluntary isometric contraction

No differences in MVIC were observed between individuals with PFP and asymptomatic controls when measured as normalised peak torque (N⋅m⋅kg^−1^) (*Condition effect: F* = *3.74,*

*p* = *0.069*; Table 1). However, overall participants exerted greater torque during the multi-joint exercise (*Exercise effect: F* = *31.03, p* < *0.001, Mean difference; MD* = *1.44 Nm/kg, 95% Confidence Interval; CI [0.897, 1.98]*).

### Motor unit recruitment thresholds

MU recruitment thresholds were comparable across muscles, groups, exercises, and torque levels (*Condition* × *Exercise* × *Muscle* × *Torque level interaction: F* = *0.86, p* = *0.461*), confirming that discharge rate comparisons were not confounded by differences in recruitment thresholds.

### Discharge rate

**Fig. 2:**
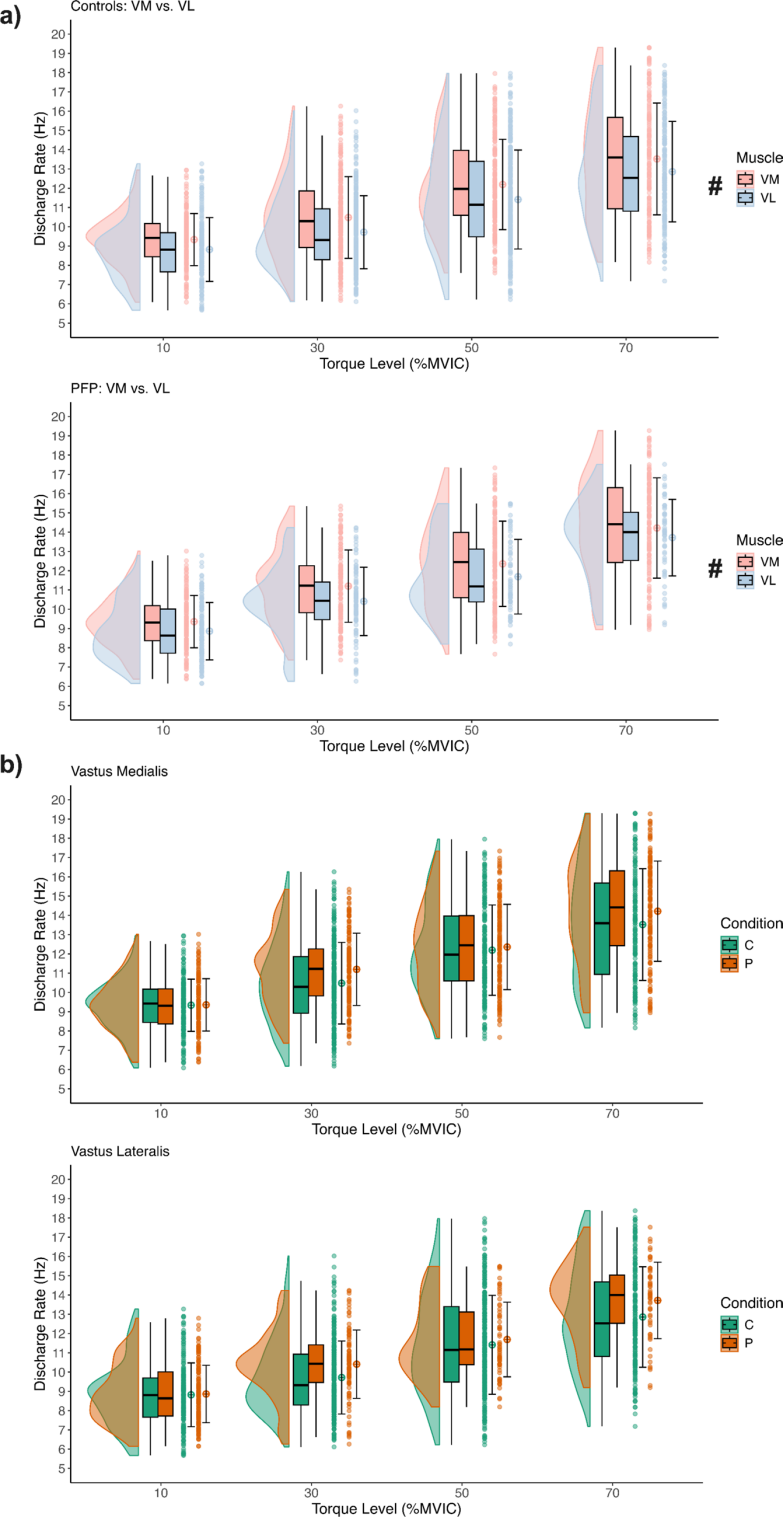
Panel **a** depicts the discharge rate at four torque levels (10%, 30%, 50%, and 70% MVIC) for the vastus medialis and vastus lateralis muscles for both the control (first graph) and the PFP group (second graph). In this panel, the distributions are illustrated with half‑violin plots accompanied by embedded boxplots and individual data points; the vastus medialis is shown in pink and the vastus lateralis in light blue. A significant Condition × Muscle interaction is evident, with post‑hoc comparisons (#, p < 0.0001 for both) indicating a higher contribution from the vastus medialis across conditions. Panel **b** presents the discharge rate for each muscle separately, the first graph for the vastus medialis and the second for the vastus lateralis, comparing Control and PFP groups. In these graphs, Control is depicted in green and PFP in orange, and no significant differences in neural drive were observed between the groups for either muscle. In both panels, the mean is displayed as a circle with error bars representing ± SD. For both panels, the data are pooled across single-joint and multi-joint exercises

A significant *Condition* × *Muscle interaction* indicated that both groups had higher discharge rates in the VM compared to the VL muscle (*F* = *6.15, p* = *0.014, PFP: MD* = *0.85 Hz, 95% CI [0.51, 1.19], Controls: MD* = *0.46, 95% CI [0.24, 0.68]*), demonstrating a consistent muscle-specific firing pattern across groups (Fig. 2a). No overall difference in discharge rate was found between those with and without PFP (*Condition effect: F* = *0.03, p* = *0.87*), suggesting comparable neural drive to the knee extensor muscles across groups (Fig. 2b). Similarly, discharge rate did not differ significantly between exercise types (Exercise effect: *F* = *1.66, p* = *0.20*), with no significant differences between exercises for either condition (Condition × Exercise: *F* = *0.13, p* = *0.72*) or muscle (Exercise × Muscle: *F* = *0.07, p* = *0.79*).

### Discharge rate variability

**Fig. 3:**
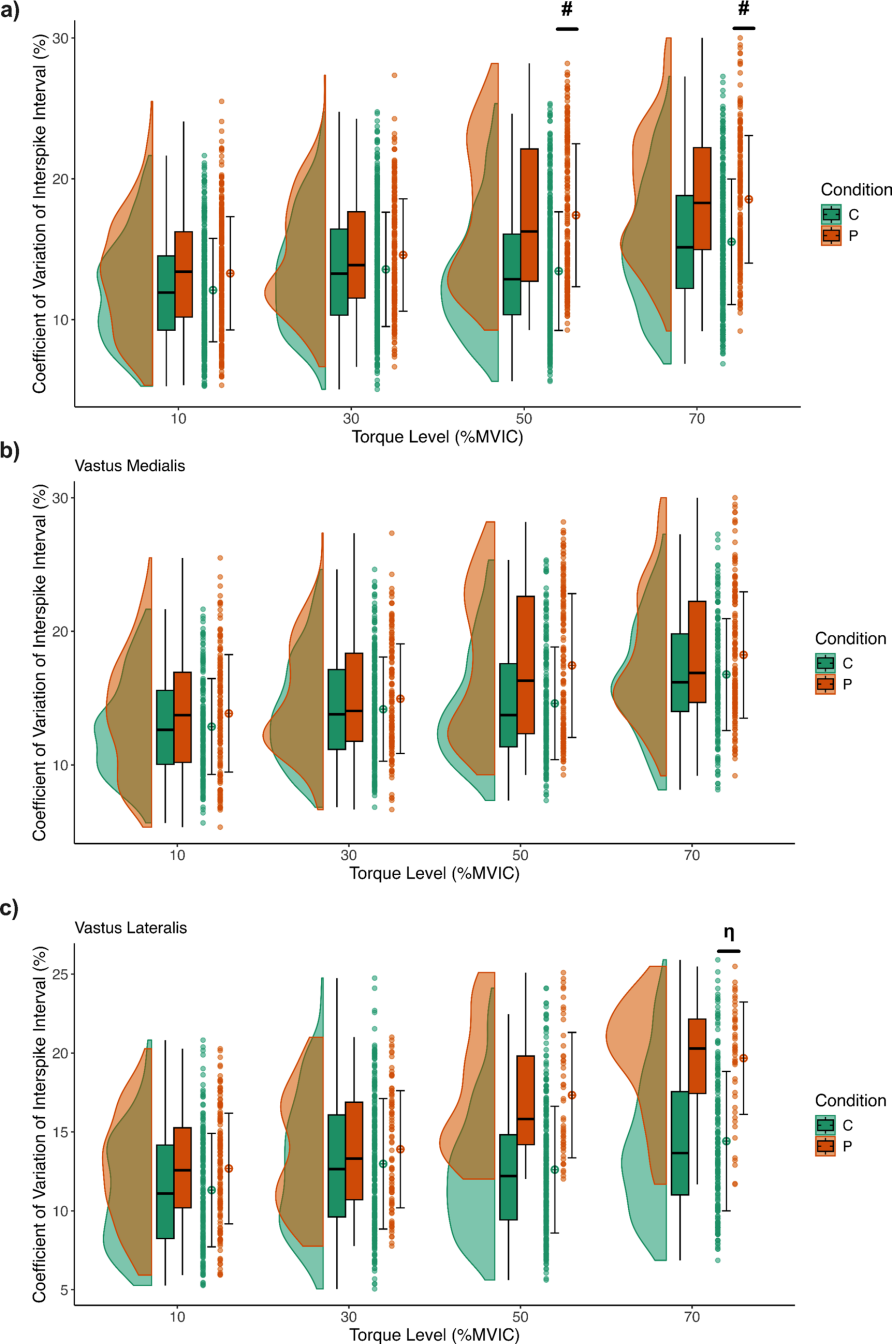
Coefficient of Variation (CoV) of interspike intervals at four torque levels (10%, 30%, 50%, and 70% MVIC) for Control (green) and PFP (orange) groups, pooled across both single‑ and multi‑joint exercises. Panels **a**, **b**, and **c** display half‑violin distributions with embedded boxplots, individual data points, and the mean (circle) ± SD for the combined vasti muscles, vastus medialis, and vastus lateralis, respectively. In panel (**a**), the # symbols (post‑hoc results of the Condition × Torque Level interaction) at 50% (p = 0.031) and 70% (p = 0.0283) reveal higher discharge rate variability in the PFP group at these higher force levels. In panel (**b**), no differences in discharge rate variability were observed for the vastus medialis across groups. In panel (**c**), the η symbol (post‑hoc result of the Condition × Muscle × Torque Level interaction, p = 0.0049) indicates significantly higher discharge rate variability for the vastus lateralis muscle in the PFP group

Discharge rate variability (CoViSi) was significantly higher in those with PFP compared to asymptomatic controls during the knee extension isometric contractions at higher torque levels (50% and 70% MVIC; *Condition* × *Torque Level interaction: F* = *12.16,* *p* < *0.0001; MD* = *− 3.32%, 95% CI [− 6.43, − 0.21] and MD* = *− 3.40%, 95% CI [− 6.54, − 0.24]*), indicating increased variability in MU firing in individuals with PFP under greater loads (Fig. 3a). Further analysis indicated that at 70% MVIC, discharge rate variability was particularly elevated in the VL muscle for those with PFP compared to controls (*Condition* × *Muscle* × *Torque Level interaction: F* = *5.04, p* = *0.0018; MD* = *− 4.92%, 95% CI [− 8.93, − 0.92]*), while no significant differences were observed in the VM muscle (Fig. 3b, c). Additionally, discharge rate variability was overall significantly higher for people with PFP compared to controls during the knee extension isometric contractions (*Condition effect: F* = *6.91,*

*p* = *0.017, MD* = *− 2.31%, 95% CI [− 4.16, − 0.467]*). Discharge rate variability did not differ significantly between exercise types (*Exercise effect: F* = *1.57, p* = *0.22*). There were also no significant two-way interactions between exercise type and condition or muscle (*Condition* × *Exercise: F* = *0.006, p* = *0.94; Exercise* × *Muscle: F* = *1.05, p* = *0.31*). The three-way interaction (*Condition* × *Exercise* × *Muscle: F* = *3.10, p* = *0.08*) was likewise not significant, further indicating no differences in discharge rate variability between conditions across exercises and muscles.

### Torque steadiness

**Fig. 4:**
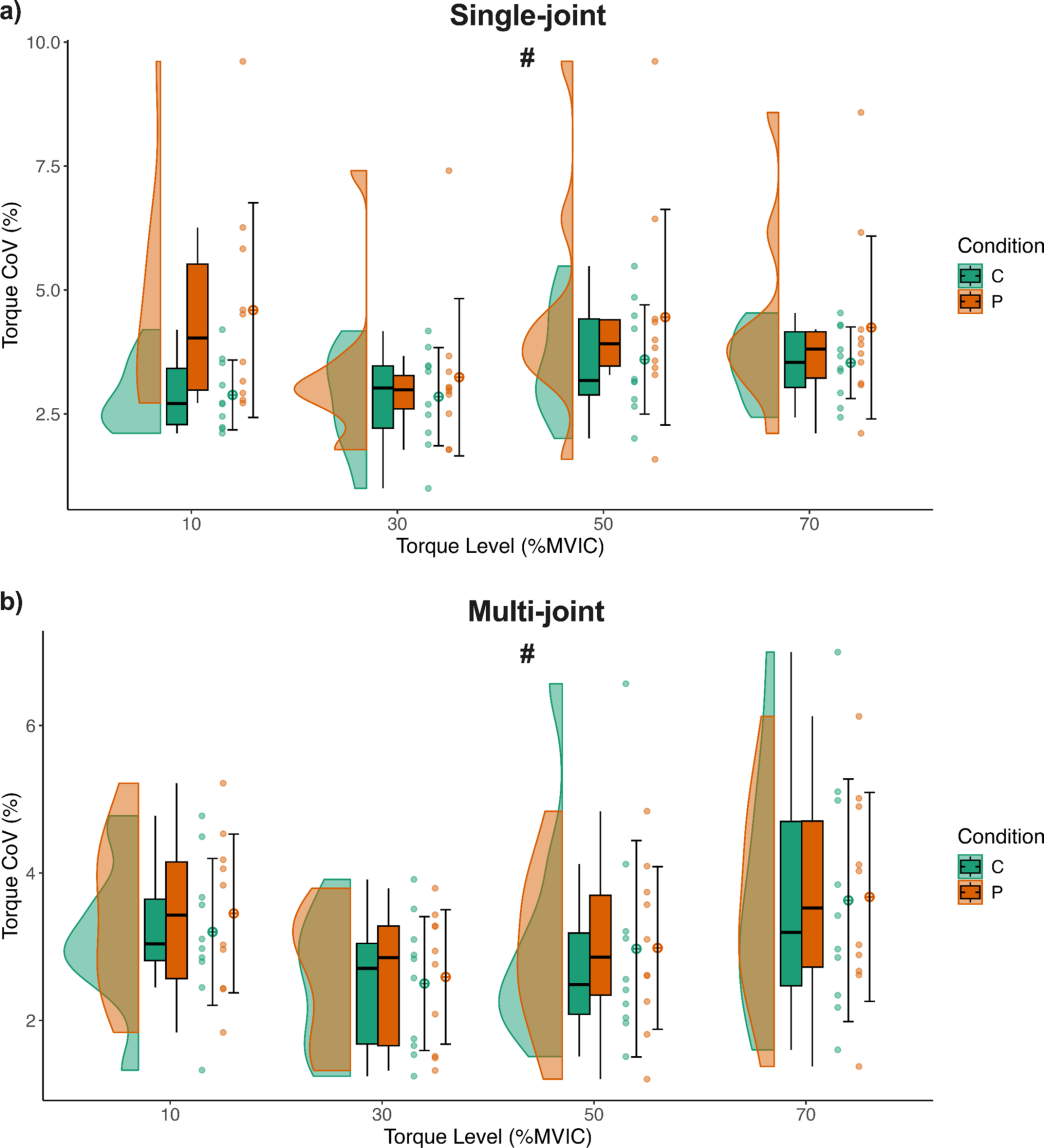
Mean ± SD values of the torque Coefficient of Variation (CoV) at four force levels (10%, 30%, 50%, and 70% MVIC) for Control (C) and PFP (P) groups, shown separately for single-joint (**a**) and multi-joint (**b**) exercises. Each panel displays raincloud plots (half‑violin distributions with embedded boxplots), individual data points, and the mean (circle) ± SD for each force level. The # symbol indicates the post-hoc result (p = 0.001) of the significant Condition × Exercise interaction, demonstrating that knee extensor steadiness was significantly worse in the PFP group during the single‑joint exercises compared to the multi‑joint exercises. A few participants with PFP showed higher torque variability values; these were verified as valid observations and retained in the analysis, representing genuine inter-individual differences in torque steadiness rather than measurement error. Residual outliers were identified and excluded based on Cook’s distance (> 4 SD) prior to analysis

Individuals with PFP exhibited reduced knee extensor isometric torque steadiness during the single-joint exercise compared to the multi-joint exercise, suggesting that torque steadiness in PFP individuals is particularly compromised during the single-joint exercise (*Condition* × *Exercise interaction: F* = *5.42, p* = *0.022, MD* = *− 0.96%, 95% CI [− 1.604, − 0.311]*; Fig. 4a, b).

### Cross-correlation

#### Peak cross-correlation

**Fig. 5:**
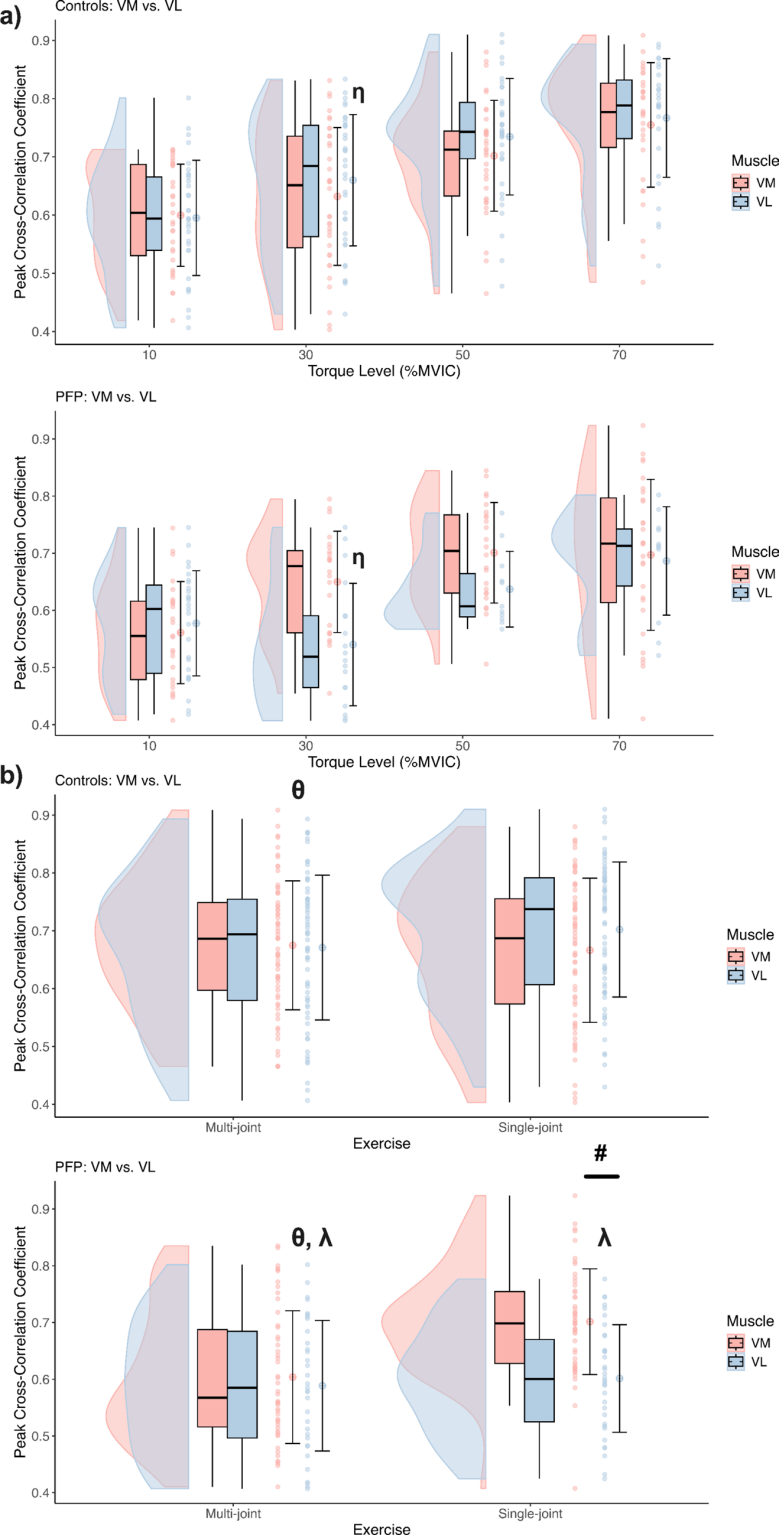
Panel **a** shows peak cross-correlation coefficients at four torque levels (10%, 30%, 50%, and 70% MVIC) for the vastus medialis (pink) and vastus lateralis (light blue) in Controls (top) and PFP (bottom). Each distribution is presented as a half-violin plot with embedded boxplots, individual data points, and the mean (circle) ± SD. The η symbol (p = 0.045, a post‑hoc result of the Condition × Muscle × Torque Level interaction) indicates that, at 30% MVIC (pooled across exercises), individuals with PFP exhibited lower cross-correlation values in the vastus lateralis, reflecting a reduced VL contribution compared to controls. Panel **b** shows peak cross-correlation coefficients for the multi‑joint vs. single‑joint exercises for the same muscles in Controls (top) and PFP (bottom). Cross-correlation coefficients are averaged across torque levels. The θ symbol (p = 0.049, a post‑hoc result of the Condition × Exercise interaction) indicates that, during the multi‑joint exercise, individuals with PFP exhibited lower cross‑correlation values (reduced vasti muscle contribution to torque) compared to controls. The λ symbol (p < 0.0001, also a post-hoc result from the Condition × Exercise interaction) shows an increase in cross‑correlation in the PFP group during the single‑joint exercise compared to the multi-joint exercise. The symbols η, θ, and λ are presented twice to denote significant pairwise comparisons from post-hoc tests at their respective locations in the figure. Lastly, the # symbol (p < 0.0001, post-hoc result of the Condition × Exercise × Muscle interaction) highlights that vastus medialis cross‑correlation was higher than vastus lateralis in the single‑joint condition, suggesting a greater vastus medialis contribution to torque

A significant *Condition* × *Muscle* × *Torque level interaction* revealed that at 30% MVIC, the control group demonstrated a higher peak of cross-correlation of the VL neural drive to torque, compared to the individuals with PFP *(F* = *4.87, p* = *0.002, MD* = *0.13, 95% CI [0.002, 0.267])* (Fig. 5a)*.* Furthermore, a significant *Condition* × *Exercise interaction* indicated that the PFP group relied more heavily on their knee extensors during single-joint exercises than during multi-joint exercises, whereas no similar difference was found for the control group (*F* = *8.76, p* = *0.003, PFP: MD* = *-0.063, 95% CI [− 0.1, − 0.03]; Control: MD* = *− 0.02, 95% CI [− 0.04, 0.01]*) (Fig. 5b, θ symbol), likely indicating a compensatory strategy adopted by individuals with PFP to accomplish the task. Subsequent analysis demonstrated that this increased contribution was predominantly from the VM muscle in the PFP group (*Condition* × *Exercise* × *Muscle interaction: F* = *11.24,*

*p* < *0.001, MD* = *− 0.10, 95% CI [− 0.15, − 0.05]*) (Fig. 5b, bottom graph, # symbol). Additionally, the *Condition* × *Exercise interaction* showed that during multi-joint exercises, the PFP group had significantly lower cross-correlation values compared to the asymptomatic controls *(MD* = *0.087, 95% CI [0.005, 0.17])* (Fig. 5b, bottom graph, λ symbol).

#### Cumulative peak cross-correlation

**Fig. 6:**
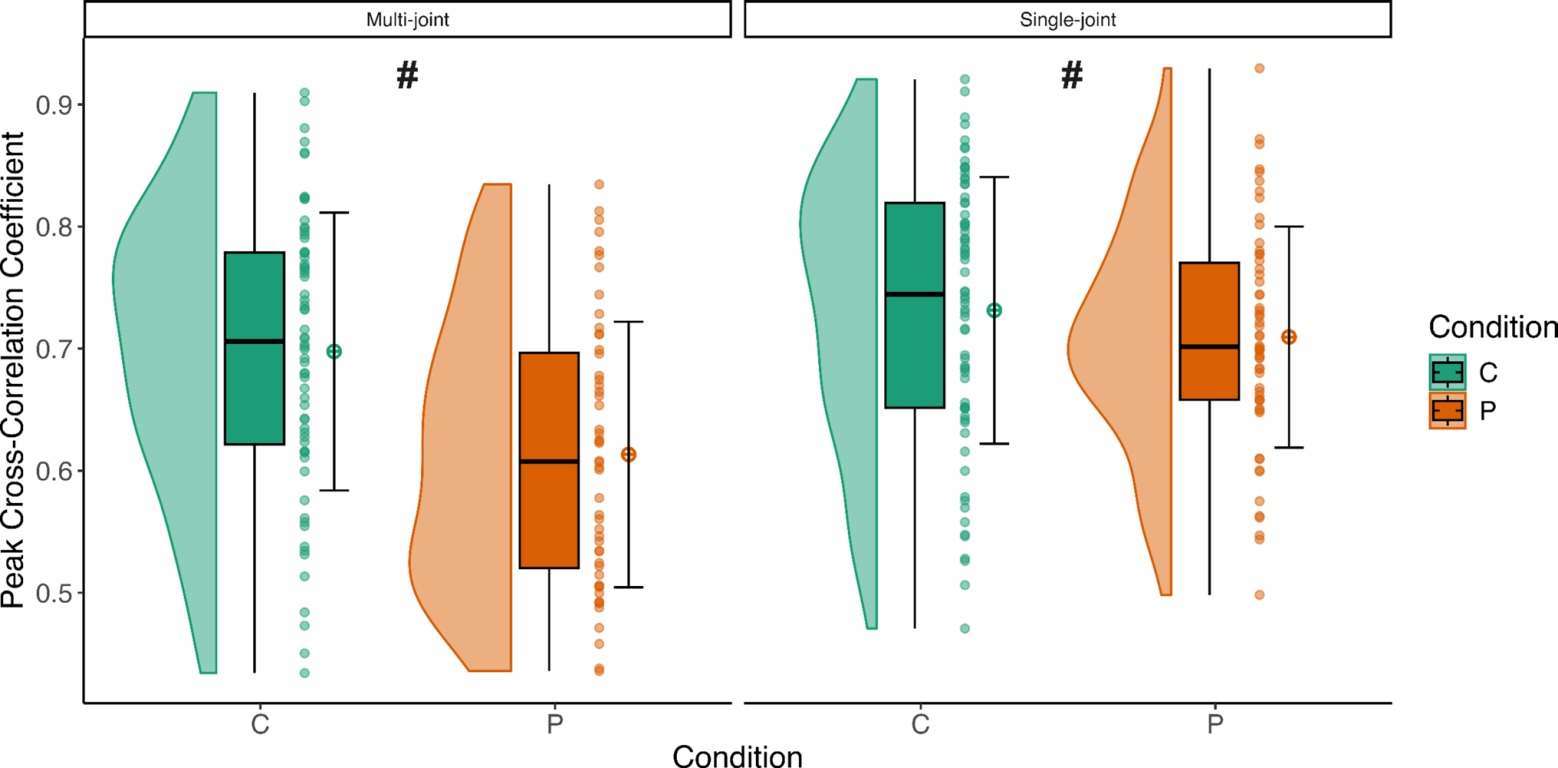
Cumulative Spike Train Peak Cross-Correlation Coefficient for knee extensor muscles in the Control (green) and PFP (orange) groups across two exercise conditions (single-joint versus multi-joint). Each panel displays half‑violin distributions with embedded boxplots, individual data points, and the mean (circle) ± SD. The values shown represent the average across all contraction intensities. The # symbols (post‑hoc results of the Condition × Exercise interaction) indicate that, for both the Control and the PFP groups, the cross-correlation values were higher during the single-joint exercise compared to the multi-joint exercise, demonstrating a greater knee extensor contribution to torque in the single-joint exercise condition (p = 0.029 and p < 0.0001)

The *Condition* × *Exercise interaction* indicated that both groups demonstrated a greater knee extensor contribution to torque during single-joint exercises (*F* = *10.85, p* = *0.0012, PFP: MD* = *− 0.079, 95% CI [-0.12, -0.04]; Controls: MD* = *− 0.04, 95% CI [− 0.08, − 0.003]*) (Fig. 6), likely reflecting task-specific neuromuscular adjustments or compensatory strategies adopted to perform single joint tasks.

### Neuromechanical delay

Analysis by individual muscle revealed a significant *Condition* × *Exercise interaction*, showing that those with PFP had a shorter NMD during single-joint exercises compared to multi-joint exercises (*F* = *6.60, p* = *0.011; MD* = *67.16 ms, 95% CI [27.23, 107.1]*), potentially indicating neuromuscular adjustments specific to task demands. More specifically, the NMD in the PFP group was 284 ms *95% CI [242, 325]* during single-joint exercises and 351 ms *95% CI [312, 389]* during multi-joint exercises. In the control group, the NMD was 329 ms, *95% CI [294, 365]* during single-joint exercises and 350 ms, *95% CI [317, 383]* during multi-joint exercises. No significant differences were observed in NMD between muscles across conditions (*Condition* × *Muscle interaction: F* = *2.61, p* = *0.108*), suggesting similar temporal coordination in MU firing patterns across both groups. There was also no significant *Condition × Exercise × Muscle* interaction (*F* = *1.35, p* = *0.25*), indicating no differences in NMD between groups across exercises and muscles. Analysis of total NMD (without separating muscles) showed a significant *Condition* × *Torque Level interaction*, with both groups demonstrating a reduction in NMD as torque levels increased (*F* = *2.96, p* = *0.035*). However, no significant overall differences in total knee extensor NMD were found between the PFP and control groups (*Condition effect: F* = *2.88, p* = *0.107*), indicating comparable delays between MU firing and torque production. Similarly, there was no significant *Condition × Exercise × Torque Level *interaction (*F* = *1.38, p* = *0.251*), further indicating no group differences at specific torque levels across exercises.

### Pain intensity

**Fig. 7:**
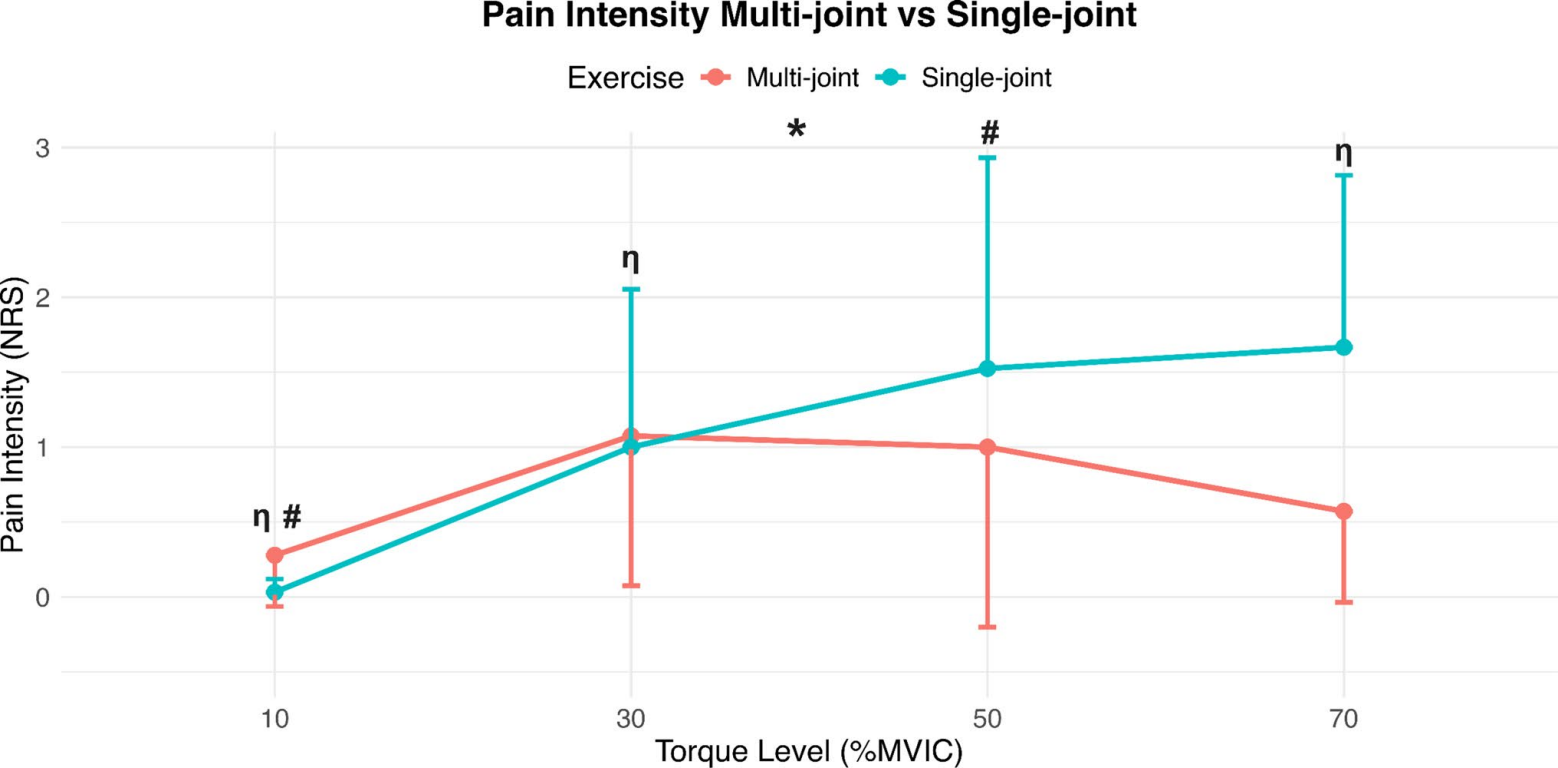
Mean ± SD values of pain intensity (Numeric Pain Rating Scale) across two exercise conditions and four force levels (%MVIC: 10%, 30%, 50%, and 70%) in participants with patellofemoral pain (PFP). Single‐joint exercises (turquoise line) elicited higher pain intensity than multi‐joint exercises (red line), as indicated by a significant main effect of exercise (*). The other two symbols reflect post-hoc results of the exercise x torque level interaction at an alpha level of 0.05. In the single‐joint condition, post‑hoc comparisons revealed that pain intensity at 50% MVIC was significantly different from that at 10%MVIC (p < 0.01, denoted by #) and pain intensity at 70% MVIC was significantly elevated compared to the 10% and 30%MVIC target torque levels (p < 0.0001 and p = 0.016, respectively, denoted by η)

A significant exercise effect was observed, with higher pain intensity during single-joint exercises compared to multi-joint exercises in individuals with PFP (*Exercise effect: F* = *4.57, p* = *0.037, MD* = *− 0.26, 95% CI [− 0.503, − 0.0161];* Fig. 7). Interestingly, as revealed by the significant* Exercise × Torque Level* interaction, this difference was primarily driven by higher pain intensity scores at higher force levels (50% and 70% MVIC) in single-joint exercises, whereas pain intensity remained relatively stable across force levels in the multi-joint exercise condition (*Exercise x Torque level interaction: F* = *3.351, p* = *0.026*; Fig. 7)*.* Specifically, at 70% MVIC, pain intensity was significantly higher compared to both 10% and 30% MVIC (*MD* = *− 1.54, 95% CI [− 2.411, − 0.6697]*, *MD* = *− 0.60, 95% CI [− 1.355, 0.1594]*, respectively), while at 50% MVIC, pain was significantly higher only when compared to 10% MVIC (*MD* = *− 1.12, 95% CI[− 1.880, − 0.3656]*).

#### Associations between outcome variables

To better understand the observed findings in the PFP group, a correlation analysis was performed between all outcome variables that showed significant differences. This analysis revealed significant associations between self-reported and objective measures in the PFP group during the single-joint exercise, where most differences were observed. More specifically, mean pain intensity correlated positively with mean torque CoV (*r* = *0.66,* *p* = *0.04*; both averaged across %MVIC levels), as well as with torque CoV at 30% MVIC (*r* = *0.78, p* = *0.008*) and 70% MVIC (*r* = *0.68, p* = *0.03*). These findings suggest that individuals experiencing higher pain levels exhibited worse torque steadiness, particularly at moderate to higher torque levels. Additionally, the Kujala score showed a significant negative correlation with mean discharge rate variability (*r* = *− 0.75, p* = *0.032*; averaged across %MVIC levels), indicating that individuals with more impaired knee function had increased MU discharge variability. All results from the correlation analyses, including non-significant associations, are provided in Supplemental File 2.

## Discussion

In this study, we compared MU behaviour of the vasti muscles and knee extensor torque steadiness during single-joint and multi-joint tasks between young physically active individuals with PFP and asymptomatic controls. Overall, neural drive (i.e., mean discharge rate) to the VM and VL muscles was similar between groups for both exercises. However, individuals with PFP exhibited reduced torque steadiness specifically during the single-joint task. This reduction in torque steadiness was accompanied by heightened MU discharge rate variability (CoViSi) at higher torque levels (50–70% MVIC), particularly at 70% MVIC in the VL muscle. Both groups demonstrated a higher contribution (MU firing-torque relationship) of the vasti muscles during the single-joint compared to the multi-joint tasks; however, in the PFP group, this was primarily driven by increased VM muscle contribution (MU firing-torque cross-correlation). Notably, during the multi-joint task, cross-correlation values between MU firing and torque were lower in the PFP group compared to controls. Additionally, people with PFP exhibited shorter NMDs during single-joint exercises. These findings likely indicate compensatory neuromuscular strategies adopted by individuals with PFP to successfully perform the tasks. Alternatively, they may reflect pain-induced changes in motor control, where nociceptive input alters synaptic drive to the motor neuron pool, modifying the neural control of the vasti muscles (Hug et al. 2025). Therefore, multi-joint exercises may mask neuromuscular deficits commonly observed at higher forces during single-joint exercises in individuals with PFP.

### Vastus medialis and lateralis firing properties in people with PFP

The ratio of VM to VL activation, typically assessed using bipolar sEMG, has previously been used to evaluate the relative contribution of each muscle during different exercises, but results have been inconsistent (Smith et al. 2009; Spairani et al. 2012; Stensdotter et al. 2007). These discrepancies are likely due to methodological factors, including electrode placement sensitivity and potential crosstalk. In this study, we utilised high-density surface EMG (HDsEMG), an established method offering improved spatial resolution, allowing detailed analysis of MU firing properties. The HDsEMG findings from the current study provide clearer insights into the differences in neural drive to the VM and VL muscles during single- and multi-joint exercises, overcoming previous methodological limitations and contributing a valuable understanding regarding muscle-specific recruitment strategies in individuals with PFP.

Despite the increasing use of HDsEMG, research on MU behaviour in individuals with PFP remains limited. To date, only one study has investigated MU firing rates in this population, reporting that VL firing rates are increased in women with PFP when they perform an isometric knee extension contraction at 10% MVIC, while VM firing rates remained similar between women with and without PFP (Gallina et al. 2018). In line with these findings, the current study also found that VM discharge rates were consistently higher than those of VL across both exercises and in both groups. Although we did not observe significant overall differences in mean discharge rates (i.e., neural drive) between groups, variability in MU firing behaviour may nonetheless influence joint mechanics and contribute to pain through altered force control and joint loading. We identified distinct alterations in the VL muscle behaviour within the PFP group, characterised by increased discharge rate variability (CoViSi) during more demanding contractions (70% MVIC). This temporal inconsistency in MU firing can compromise the smoothness of force output and alter the distribution of forces acting on the patella, potentially affecting the coordinated VM-VL activation thought to be important for maintaining proper patellar tracking within the trochlear groove (Loudon 2016; Powers et al. 2017). These alterations may increase mediolateral patellar displacement and mechanical stress on periarticular tissues. Given the substantial role of VL in force generation as the largest quadriceps muscle (Biondi and Varacallo 2025), this elevated discharge variability likely reflects either instability in MU firing or compensatory neuromuscular strategies employed to meet the increased force demands at 70% MVIC. However, these adaptations might also contribute to patellar maltracking and explain the higher pain intensity reported during these challenging contractions (Lutz et al. 1993; Powers et al. 2003).

While our findings are comparable with those of (Gallina et al. 2018), slight discrepancies may stem from methodological differences between the studies. First, the previous study included only female participants, whereas our sample comprised both sexes, which may have introduced differences in neuromuscular control strategies and MU behaviour, given the documented sex-related variations in vasti muscle activation patterns (Dimmick et al. 2022; Lulic-Kuryllo and Inglis 2022; Peng et al. 2018). Second, differences in knee joint angle during testing (e.g., 90° vs. 60° of knee flexion) can alter muscle length–tension relationships and, consequently, modify MU recruitment and discharge behaviour (Valenčič et al. 2024). Finally, variations in sample size and electrode placement may influence the number, spatial distribution, and detectability of MUs, thereby affecting the characterisation of MU properties. These factors highlight the complexity of neuromuscular adaptations in people with PFP and the need for further research to understand how task constraints and individual characteristics shape MU recruitment and firing patterns.

In addition to these findings, it is important to consider how other quadriceps components may contribute to knee extensor function and neuromuscular control. Although we focused on the VM and VL muscles, it is important to consider the remaining quadriceps components, the rectus femoris (RF) and vastus intermedius (VI). All four muscles act as knee extensors, with the RF also contributing to hip flexion due to its biarticular nature (Watanabe and Akima 2011). However, it has been suggested that the quadriceps components possess different force-production properties and are not functionally identical. Recent evidence supports this view, showing distinct neuromuscular control strategies among the quadriceps. During a bipedal squat, common synaptic input differs between the biarticular RF and the monoarticular vasti, with significantly higher coherence between VM-VL than between RF-VL or RF-VM, indicating divergent motor control between biarticular and monoarticular muscles (Maudrich et al. 2022). Moreover, RF activation itself appears heterogeneous, with regional differences in myoelectric activity across its proximal and distal portions during squat exercises (de Souza et al. 2017). The VI, while monoarticular, exhibits lower activation than the superficial vasti and a joint-angle-dependent pattern of activation that is not consistent with the other quadriceps muscles (Akima and Saito 2013; Watanabe and Akima 2011). Taken together, these findings indicate that RF and VI are likely controlled differently from VM and VL, reflecting task- and architecture-specific neuromuscular strategies. However, caution is needed when interpreting these findings, as they are derived from asymptomatic individuals using EMG amplitude as a measure of muscle activity rather than analyses at the MU level. Future studies should explore whether similar differential activation patterns and MU behaviour occur in people with PFP, where pain-related adaptations may further influence quadriceps coordination.

### Knee extension versus leg press

While the neural drive to the vasti muscles was similar between groups and exercises, notable differences between the two exercises were observed in the PFP group for the other outcome variables. Specifically, single-joint exercises led to a greater increase in pain intensity, particularly as task demands increased. This may be attributed to the greater mechanical stress imposed on the patellofemoral joint in single-joint exercises, where reduced joint compression and increased shear forces could lead to excessive joint surface movement, exacerbating irritation of pain-sensitive structures such as the subchondral bone, synovium, and retinaculum (Lutz et al. 1993).

The differences observed between exercises may also be explained by the nature of the leg press as a multi-joint exercise, which recruits additional muscle groups, such as the hip extensors, allowing for compensatory strategies and greater joint stability, which may account for the better torque steadiness performance (Kwon et al. 2013). This was further supported by lower cross-correlation values observed in both groups, indicating a reduced reliance on the vasti muscles for torque generation during the multi-joint task. Additionally, despite similar discharge rates between single-joint and multi-joint tasks, participants exerted higher force during the multi-joint exercise task, which was expected as more muscle groups were recruited to perform the task. In the PFP group, the VM showed an increased contribution (MU firing-torque relationship) from the multi-joint to the single-joint task, and a decrease in the NMD. This pattern suggests a neuromuscular adjustment to maintain performance and may contribute to the impaired force control observed during the single-joint exercise.

This study also highlighted neuromuscular impairments during single-joint tasks in individuals with PFP, particularly in conditions of increased mechanical demand. Notably, reduced torque steadiness was associated with higher pain intensity, with individuals experiencing greater pain showing worse torque steadiness. This decline in torque steadiness was accompanied by increased MU discharge rate variability, particularly at higher torque levels (50–70% MVIC), and of the VL muscle, suggesting impaired motor control under greater mechanical demands. Increased variability in discharge rate may indicate deficits in synaptic input regulation, potentially due to maladaptive changes in corticospinal drive or altered proprioceptive feedback from the knee joint.

These findings align with the concept that force control is influenced by variability in MU activity, which can be quantified using the coefficient of variation of the smoothed firing rate signal (CoViSi). CoViSi reflects the variability in neural drive and has been identified as a key determinant of force steadiness (Farina and Negro 2015). Increased CoViSi, particularly at higher contraction intensities, suggests greater fluctuations in synaptic input, contributing to reduced torque steadiness. Given that individuals with PFP exhibited increased MU discharge rate variability alongside reduced torque steadiness, it is plausible that deficits in neural drive consistency contribute to impaired motor control in this population. These findings support the idea that neuromuscular deficits in PFP extend beyond VM and VL activation imbalances, highlighting broader impairments in neural drive regulation and stability. The significant correlations between torque variability, discharge variability, self-reported pain, and knee function further reinforce the link between altered neuromuscular control and symptom severity.

### Potential mechanisms underlying neuromuscular impairments in PFP

It is well known that pain can alter movement patterns and muscle recruitment strategies (Hodges and Tucker 2011). Pain likely impairs muscle force control through altered sensory input and central processing (Ager et al. 2020; Proske and Gandevia 2012). Structural changes in brain regions responsible for proprioception or modifications in muscle composition may further contribute to these deficits (Pijnenburg et al. 2014; Sterling et al. 2001). Such alterations in sensory integration can influence the efferent response, leading to adaptations in muscle recruitment strategies during contractions.

Impairments in force steadiness are closely linked to MU behaviour. Increased MU recruitment and firing rates have been observed in early knee osteoarthritis (Ling et al. 2007) and women with PFP (Gallina et al. 2018). Experimentally induced knee pain may also affect force steadiness and modify MU discharge patterns, reinforcing the role of pain as the trigger for neuromuscular adaptations (Poortvliet et al. 2019; Rice et al. 2015).

Neural drive predominantly operates within the low-frequency band (< 10 Hz), reflecting common synaptic input to the MU pool, which is crucial for force generation (Farina et al. 2014; Negro et al. 2009). Increased amplitude fluctuations in this range, observed in pain conditions, suggest greater variability in synaptic input to α motor neurons. Given their role in integrating proprioceptive and nociceptive signals, the impact of musculoskeletal pain on force control may differ across individuals and pain types (Hug et al. 2023). This mechanism may explain why most of the observed neuromuscular impairments in individuals with PFP were more evident during single-joint exercises and higher-intensity contractions, as these conditions were associated with increased pain levels.

### Methodological considerations

This study has some methodological limitations that should be considered when interpreting the findings. The sample size was relatively small and consisted primarily of male and female young physically active people, which may not fully represent the broader population of individuals with PFP, particularly those who are less physically active or from different age groups. Being physically active can influence the neural and muscular characteristics of the vasti muscles. Some studies have shown that strength or endurance training can modify MU behaviour and muscle properties, with strength training reducing discharge rate variability and improving torque steadiness (Vila-Chã and Falla 2016), and resistance training altering the sensorimotor control of the vasti muscles (Wong and Ng 2010). Chronic endurance training has been also shown to result in improved muscle tissue composition and altered MU recruitment characteristics of the VL, reflected by lower echo intensity and larger MU action potentials compared with sedentary individuals (Dimmick et al. 2018). However, findings are inconsistent, as long-term resistance-trained men show similar MU discharge characteristics to untrained individuals (Škarabot et al. 2023), and a recent meta-analysis found no overall change in MU behaviour after resistance training (Elgueta-Cancino et al. 2022). Importantly, the findings of these studies are based on asymptomatic participants under controlled training conditions rather than in athletes engaged in specific sports, and it remains unclear whether similar differences between trained and untrained individuals would be observed in people with PFP. Pain and disuse may further modify neuromuscular and morphological adaptations, potentially amplifying these effects. Consequently, while our physically active participants demonstrated significant neuromuscular impairments, it remains uncertain whether sedentary individuals with PFP would show similar, more pronounced, or different patterns of MU dysfunction. Future research should examine how these factors interact to influence MU behaviour and muscle function in this population. Given that neuromuscular adaptations in PFP may vary depending on factors such as training background, activity level, and chronicity of symptoms, the findings should be interpreted with caution when generalising to other populations. Nevertheless, our results demonstrate that neuromuscular impairments are evident even in individuals with PFP who maintain regular training and high physical activity levels, particularly during single-joint exercises and under higher mechanical demands. This suggests that even in highly active individuals, neuromuscular deficits are present, reinforcing the need to address these impairments in rehabilitation.

Another limitation relates to the contraction intensities used. Contractions were limited to 70%MVIC because reliable decomposition of MU activity from HDsEMG becomes increasingly challenging at higher intensities due to signal superposition (Negro et al. 2016). It remains unclear how MU behaviour and torque steadiness might differ at intensities exceeding this threshold, which should be investigated in future research. Moreover, significant group differences in the peak cross-correlation coefficient (per muscle analysis) were observed only at 30% MVIC. This pattern is most likely explained by methodological rather than physiological factors. Specifically, the decomposition quality and the number of identified MUs were highest at this contraction intensity, which increased the reliability of the cross-correlation estimates and the statistical power to detect group differences. At very low and high force levels, fewer MUs could be reliably decomposed, reducing the stability of the estimates and the ability to detect additional effects. This interpretation is supported by the MU counts, as shown in Supplemental File 3, where MU yield at higher intensities, particularly for the VL in the PFP group during the single-joint exercise, was markedly reduced compared to the controls. Therefore, we believe that the observed difference at 30% MVIC reflects a real neuromuscular adaptation, but that technical limitations related to MU yield at other contraction intensities may have masked further differences. As this was a pilot study with a relatively small sample, future studies including larger cohorts and improved decomposition performance across contraction levels are expected to increase the sensitivity of cross-correlation measures to detect between-group differences throughout the full range of torque levels.

### Clinical implications and future directions

The results support the use of multi-joint exercises as an initial rehabilitation approach, as they are likely to produce less pain and are associated with better torque steadiness. Multi-joint exercises also elicit comparable vasti muscle activation, suggesting they can effectively engage the quadriceps while minimising pain. However, they may allow for greater neuromuscular adjustments, potentially masking impairments that become more evident during single-joint exercises. Our findings show that individuals with PFP exhibit more pronounced neuromuscular impairments during single-joint exercises, particularly at higher force levels. Previous research has shown that multi-joint exercises promote more balanced initial quadriceps activation compared with single-joint tasks (Stensdotter et al. 2003), which may explain their preference early in rehabilitation. However, long-term evidence indicates that both approaches result in comparable functional outcomes, with some reports of fewer residual complaints after single-joint training (Witvrouw et al. 2004). Therefore, single-joint exercises should not be overlooked, as they can complement multi-joint exercises by addressing deficits in motor control and torque steadiness that may remain undetected in multi-joint tasks. This perspective aligns with current clinical guidelines indicating that weight-bearing and non–weight-bearing quadriceps exercises impose different patterns of patellofemoral joint loading, each offering distinct advantages that can influence patient outcomes (Willy et al. 2019). Rehabilitation programs should incorporate strategies to address these deficits and restore optimal muscle function across various tasks. Importantly, torque steadiness training may be a promising intervention, as enhancing the stability of force output could improve motor control and alleviate symptoms. Future research should examine whether improving torque steadiness leads to clinical improvements and investigate MU behaviour in larger, more diverse PFP samples.

## Conclusion

This pilot study provides novel insights into the neuromuscular impairments associated with PFP, particularly highlighting increased MU discharge rate variability and reduced torque steadiness during single-joint exercises. Future research should further investigate the underlying neurophysiological mechanisms and explore targeted interventions aimed at improving force control and reducing symptom severity in this population.

## Supplementary Information

Below is the link to the electronic supplementary material.

Supplementary file1 (DOCX 41 KB)

Supplementary file2 (DOCX 32 KB)

Supplementary file3 (DOCX 28 KB)

## Data Availability

The datasets generated and/or analysed during the current study are available from the lead author (MA) upon reasonable request.
