# Supplementary material for: Reduced knee extensor torque steadiness and increased motor unit discharge rate variability in young people with patellofemoral pain: a pilot study
Source: Eur J Appl Physiol. 2025 Dec 22;126(5):2595–615. doi: 10.1007/s00421-025-06083-8 (PMC13236768; doi:10.1007/s00421-025-06083-8)
Supplement: Supplementary file 1 — Supplementary file1 (DOCX 41 KB) [file 421_2025_6083_MOESM1_ESM.docx]

***Supplemental Statistical Results:***

***Type III Analysis of Variance from Linear Mixed Models***

**Document Overview:** This document presents the results of Type III Analysis of Variance (ANOVA) examining main effects and interactions across multiple dependent variables.

**Statistical Significance:** Significant effects or interactions (*p < 0.05*) are highlighted in **yellow with bold text**. Results are organised by dependent variable, with main effects presented first, followed by two-way, three-way, and four-way interactions where applicable.

**Abbreviations**

**CoV:** Coefficient of Variation

**CoViSi:** Coefficient of Variation of Interspike Intervals (Discharge Rate Variability)

**MVIC:** Maximum Voluntary Isometric Contraction

Variable Descriptions

**Condition:** Patellofemoral Knee Pain vs. Control

**Exercise:** Exercise type (single-joint vs. multi-joint)

**Muscle:** Specific muscle analysed (Vastus Medialis, Vastus Lateralis)

**Torque Level:** Percentage of MVIC (10%, 30%, 50%, 70%)

1. ***MVIC***

| **Effect** | **F-value** | **P-value** |
| --- | --- | --- |
| ***Main Effects*** | | |
| Condition | *3.74* | *0.069* |
| Exercise | ***31.03*** | ***<0.001*** |
| ***Two-Way Interactions*** | | |
| Condition x Exercise | *0.24* | *0.63* |

1. ***Motor Unit Recruitment Thresholds***

| **Effect** | **F-value** | **P-value** |
| --- | --- | --- |
| ***Main Effects*** | | |
| Condition | *0.98* | *0.34* |
| Exercise | ***46.39*** | ***<0.0001*** |
| Muscle | ***9.47*** | ***0.002*** |
| Torque Level | ***5103.70*** | ***<0.0001*** |
| ***Two-Way Interactions*** | | |
| Condition x Exercise | ***32.13*** | ***<0.0001*** |
| Condition x Muscle | ***15.82*** | ***<0.0001*** |
| Exercise x Muscle | *1.72* | *0.19* |
| Condition x Torque Level | ***2.43*** | ***0.06*** |
| Exercise x Torque Level | ***25.68*** | ***<0.0001*** |
| Muscle x Torque Level | *1.54* | *0.20* |
| ***Three-Way Interactions*** | | |
| Condition x Exercise x Muscle | *1.95* | *0.16* |
| Condition x Exercise x Torque Level | ***14.68*** | ***<0.0001*** |
| Condition x Muscle x Torque Level | ***4.65*** | ***0.0030*** |
| Exercise x Muscle x Torque Level | ***2.93*** | ***0.033*** |
| ***Four-Way Interactions*** | | |
| Condition x Exercise x Muscle x Torque Level | *0.86* | *0.461* |

1. ***Discharge Rate***

| **Effect** | **F-value** | **P-value** |
| --- | --- | --- |
| ***Main Effects*** | | |
| Condition | *0.03* | *0.87* |
| Exercise | *1.66* | *0.20* |
| Muscle | ***68.21*** | ***<0.0001*** |
| Torque Level | ***719.06*** | ***<0.0001*** |
| ***Two-Way Interactions*** | | |
| Condition x Exercise | *0.13* | *0.72* |
| Condition x Muscle | ***6.15*** | ***0.014*** |
| Exercise x Muscle | *0.07* | *0.79* |
| Condition x Torque Level | ***3.25*** | ***0.021*** |
| Exercise x Torque Level | ***7.50*** | ***<0.0001*** |
| Muscle x Torque Level | *0.82* | *0.48* |
| ***Three-Way Interactions*** | | |
| Condition x Exercise x Muscle | *0.01* | *0.91* |
| Condition x Exercise x Torque Level | ***3.33*** | ***0.02*** |
| Condition x Muscle x Torque Level | *0.79* | *0.50* |
| Exercise x Muscle x Torque Level | *2.12* | *0.095* |
| ***Four-Way Interactions*** | | |
| Condition x Exercise x Muscle x Torque Level | *2.60* | *0.052* |

1. ***Discharge Rate Variability (CoViSi)***

| **Effect** | **F-value** | **P-value** |
| --- | --- | --- |
| ***Main Effects*** | | |
| Condition | ***6.91*** | ***0.017*** |
| Exercise | *1.57* | *0.22* |
| Muscle | ***29.18*** | ***<0.0001*** |
| Torque Level | *153.19* | ***<0.0001*** |
| ***Two-Way Interactions*** | | |
| Condition x Exercise | *0.006* | *0.94* |
| Condition x Muscle | *2.53* | *0.11* |
| Exercise x Muscle | *1.05* | *0.31* |
| Condition x Torque Level | ***12.16*** | ***< 0.0001*** |
| Exercise x Torque Level | *9.52* | ***<0.0001*** |
| Muscle x Torque Level | *2.44* | *0.063* |
| ***Three-Way Interactions*** | | |
| Condition x Exercise x Muscle | *3.10* | *0.08* |
| Condition x Exercise x Torque Level | *1.14* | *0.34* |
| Condition x Muscle x Torque Level | ***5.04*** | ***0.0018*** |
| Exercise x Muscle x Torque Level | ***2.98*** | ***0.03*** |
| ***Four-Way Interactions*** | | |
| Condition x Exercise x Muscle x Torque Level | ***3.75*** | ***0.011*** |

1. ***Torque Steadiness (Torque CoV)***

| **Effect** | **F-value** | **P-value** |
| --- | --- | --- |
| ***Main Effects*** | | |
| Condition | *1.57* | *0.23* |
| Exercise | ***9.75*** | ***0.002*** |
| Torque Level | ***5.73*** | ***0.001*** |
| ***Two-Way Interactions*** | | |
| Condition x Exercise | ***5.42*** | ***0.022*** |
| Condition x Torque Level | *0.86* | *0.47* |
| Exercise x Torque Level | *0.99* | *0.40* |
| ***Three-Way Interactions*** | | |
| Condition x Exercise x Torque Level | *0.47* | *0.70* |

1. ***Peak Cross-Correlation (Analysis per Muscle)***

| **Effect** | **F-value** | **P-value** |
| --- | --- | --- |
| ***Main Effects*** | | |
| Condition | *4.38* | *0.05* |
| Exercise | ***23.33*** | ***<0.0001*** |
| Muscle | ***4.18*** | ***0.042*** |
| Torque Level | *62.46* | ***<0.0001*** |
| ***Two-Way Interactions*** | | |
| Condition x Exercise | ***8.76*** | ***0.003*** |
| Condition x Muscle | *17.55* | ***<0.0001*** |
| Exercise x Muscle | *1.63* | *0.20* |
| Condition x Torque Level | *2.23* | *0.084* |
| Exercise x Torque Level | *0.63* | *0.60* |
| Muscle x Torque Level | *2.25* | *0.082* |
| ***Three-Way Interactions*** | | |
| Condition x Exercise x Muscle | ***11.24*** | ***<0.001*** |
| Condition x Exercise x Torque Level | ***2.98*** | ***0.032*** |
| Condition x Muscle x Torque Level | ***4.87*** | ***0.002*** |
| Exercise x Muscle x Torque Level | *1.00* | *0.39* |
| ***Four-Way Interactions*** | | |
| Condition x Exercise x Muscle x Torque Level | *2.04* | *0.11* |

1. ***Cumulative Peak Cross-Correlation***

| **Effect** | **F-value** | **P-value** |
| --- | --- | --- |
| ***Main Effects*** | | |
| Condition | *3.53* | *0.077* |
| Exercise | ***56.00*** | ***<0.0001*** |
| Torque Level | ***38.50*** | ***<0.0001*** |
| ***Two-Way Interactions*** | | |
| Condition x Exercise | ***10.85*** | ***0.0012*** |
| Condition x Torque Level | *1.65* | *0.18* |
| Exercise x Torque Level | *2.29* | *0.079* |
| ***Three-Way Interactions*** | | |
| Condition x Exercise x Torque Level | *0.43* | *0.73* |

1. ***Neuromechanical delay (Analysis per Muscle)***

| **Effect** | **F-value** | **P-value** |
| --- | --- | --- |
| ***Main Effects*** | | |
| Condition | *1.65* | *0.212* |
| Exercise | ***23.71*** | ***<0.0001*** |
| Muscle | *1.77* | *0.19* |
| Torque Level | ***56.36*** | ***<0.0001*** |
| ***Two-Way Interactions*** | | |
| Condition x Exercise | ***6.60*** | ***0.011*** |
| Condition x Muscle | *2.61* | *0.108* |
| Exercise x Muscle | *0.28* | *0.60* |
| Condition x Torque Level | ***2.96*** | ***0.036*** |
| Exercise x Torque Level | ***3.71*** | ***0.013*** |
| Muscle x Torque Level | *0.75* | *0.52* |
| ***Three-Way Interactions*** | | |
| Condition x Exercise x Muscle | *1.35* | *0.25* |
| Condition x Exercise x Torque Level | ***8.18*** | ***<0.0001*** |
| Condition x Muscle x Torque Level | *1.47* | *0.23* |
| Exercise x Muscle x Torque Level | *2.05* | *0.11* |
| ***Four-Way Interactions*** | | |
| Condition x Exercise x Muscle x Torque Level | ***2.73*** | ***0.045*** |

1. ***Neuromechanical delay (based on cumulative analysis)***

| **Effect** | **F-value** | **P-value** |
| --- | --- | --- |
| ***Main Effects*** | | |
| Condition | *2.88* | *0.107* |
| Exercise | ***18.45*** | ***<0.0001*** |
| Torque Level | ***57.31*** | ***<0.0001*** |
| ***Two-Way Interactions*** | | |
| Condition x Exercise | *2.33* | *0.130* |
| Condition x Torque Level | ***2.96*** | ***0.035*** |
| Exercise x Torque Level | *1.59* | *0.196* |
| ***Three-Way Interactions*** | | |
| Condition x Exercise x Torque Level | *1.38* | *0.251* |

1. ***Pain Intensity***

| **Effect** | **F-value** | **P-value** |
| --- | --- | --- |
| ***Main Effects*** | | |
| Exercise | ***4.61*** | ***0.037*** |
| Torque Level | ***12.70*** | ***<0.0001*** |
| ***Two-Way Interactions*** | | |
| Exercise x Torque Level | *3.35* | *0.026* |
