# Supplementary material for: Reduced knee extensor torque steadiness and increased motor unit discharge rate variability in young people with patellofemoral pain: a pilot study
Source: Eur J Appl Physiol. 2025 Dec 22;126(5):2595–615. doi: 10.1007/s00421-025-06083-8 (PMC13236768; doi:10.1007/s00421-025-06083-8)
Supplement: Supplementary file 2 — Supplementary file2 (DOCX 32 KB) [file 421_2025_6083_MOESM2_ESM.docx]

***Supplemental Statistical Results:***

***Correlation Analysis (associations between outcome variables)***

**Document Overview:** This document presents correlation analyses examining the relationships between pain intensity, functional scores, and biomechanical variables across different torque intensities and exercise conditions. Each correlation is reported with Pearson's correlation coefficient (r) and statistical significance (p-value).

**Significant Results:** Statistically significant findings (p < 0.05) are highlighted in **yellow with bold text**.

**Data Aggregation:**

- **Mean:** Values averaged across torque intensities (10%, 30%, 50%, 70%)
- **Single-Joint:** Refers to single-joint exercise conditions
- **Multi-Joint:** Refers to multi-joint exercise conditions
- **Pooled:** Data pooled across both single-joint and multi-joint exercises

**Abbreviations**

**CoV:** Coefficient of Variation

**CoViSi:** Coefficient of Variation of Interspike Interval (measure of discharge rate variability)

**XCORR:** Cross-correlation

*Note: Torque intensities (10%, 30%, 50%, 70%) represent percentage levels of maximum voluntary contraction during testing protocols.*

***Pain Intensity vs Torque CoV (Single-Joint)***

| **Pain Intensity Level** | **10% Torque CoV** | **30% Torque CoV** | **50% Torque CoV** | **70% Torque CoV** |
| --- | --- | --- | --- | --- |
| 10% | *r = -0.14, p = 0.70* | *r = 0.48, p = 0.16* | *r = 0.02, p = 0.95* | ***r = 0.79, p = 0.006*** |
| 30% | *r = -0.07, p = 0.84* | ***r = 0.78, p = 0.008*** | *r = 0.16, p = 0.66* | ***r = 0.91, p = 0.0003*** |
| 50% | *r = 0.01, p = 0.98* | ***r = 0.91, p = 0.0002*** | *r = 0.53, p = 0.11* | ***r = 0.91, p = 0.0003*** |
| 70% | *r = -0.02, p = 0.95* | *r = 0.62, p = 0.054* | *r = 0.18, p = 0.63* | ***r = 0.68, p = 0.03*** |

**Mean Pain Intensity (Single-Joint) - Mean Torque CoV (Single-Joint): *r = 0.66, p = 0.04***

***Pain Intensity vs Torque CoV (Multi-Joint)***

| **Pain Intensity Level** | **10% Torque CoV** | **30% Torque CoV** | **50% Torque CoV** | **70% Torque CoV** |
| --- | --- | --- | --- | --- |
| 10% | *r = 0.18, p = 0.62* | ***r = 0.69, p = 0.026*** | *r = 0.06, p = 0.87* | *r = 0.58, p = 0.08* |
| 30% | *r = 0.18, p = 0.62* | ***r = 0.55, p = 0.01*** | *r = 0.24, p = 0.50* | ***r = 0.65, p = 0.041*** |
| 50% | *r = 0.45, p = 0.19* | ***r = 0.64, p = 0.046*** | *r = 0.44, p = 0.20* | ***r = 0.68, p = 0.029*** |
| 70% | *r = 0.27, p = 0.45* | *r = 0.40, p = 0.251* | *r = 0.43, p = 0.21* | *r = 0.50, p = 0.144* |

**Mean Pain Intensity (Multi-Joint) - Mean Torque CoV (Multi-Joint):** *r = 0.58, p = 0.08*

***Pain Intensity vs CoViSi (Single-Joint)***

| **Pain Intensity Level** | **10% CoViSi** | **30% CoViSi** | **50% CoViSi** | **70% CoViSi** |
| --- | --- | --- | --- | --- |
| 10% | *r = 0.70, p = 0.053* | *r = 0.55, p = 0.154* | *r = 0.21, p = 0.63* | ***r = 0.74, p = 0.036*** |
| 30% | *r = 0.70, p = 0.055* | *r = 0.64, p = 0.089* | *r = 0.25, p = 0.55* | *r = 0.32, p = 0.44* |
| 50% | *r = 0.56, p = 0.146* | *r = 0.50, p = 0.21* | *r = 0.24, p = 0.57* | *r = 0.07, p = 0.862* |
| 70% | *r = 0.68, p = 0.064* | *r = 0.46, p = 0.26* | *r = 0.64, p = 0.09* | *r = 0.17, p = 0.683* |

**Mean Pain Intensity (Single-Joint) - Mean CoViSi (Single-Joint):** r = 0.64, p = 0.086

***Pain Intensity vs CoViSi (Multi-Joint)***

| **Pain Intensity Level** | **10% CoViSi** | **30% CoViSi** | **50% CoViSi** | **70% CoViSi** |
| --- | --- | --- | --- | --- |
| 10% | *r = 0.45, p = 0.267* | *r = 0.57, p = 0.142* | *r = 0.29, p = 0.49* | *r = 0.50, p = 0.21* |
| 30% | *r = 0.51, p = 0.194* | *r = 0.30, p = 0.478* | *r = 0.54, p = 0.169* | ***r = 0.73, p = 0.038*** |
| 50% | *r = 0.65, p = 0.083* | *r = 0.27, p = 0.511* | *r = 0.51, p = 0.198* | *r = 0.62, p = 0.10* |
| 70% | *r = 0.58, p = 0.129* | *r = 0.25, p = 0.545* | *r = 0.55, p = 0.160* | *r = 0.45, p = 0.266* |

**Mean Pain Intensity (Multi-Joint) - Mean CoViSi (Multi-Joint):** *r = 0.63, p = 0.07*

***Torque CoV vs CoViSi (Single-Joint)***

| **Torque CoV Level** | **10% CoViSi** | **30% CoViSi** | **50% CoViSi** | **70% CoViSi** |
| --- | --- | --- | --- | --- |
| 10% | *r = 0.48, p = 0.227* | ***r = -0.78, p = 0.021*** | *r = -0.36, p = 0.376* | *r = 0.08, p = 0.852* |
| 30% | *r = 0.43, p = 0.29* | *r = 0.45, p = 0.26* | *r = 0.06, p = 0.89* | *r = -0.13, p = 0.76* |
| 50% | *r = -0.15, p = 0.72* | *r = 0.17, p = 0.682* | *r = 0.31, p = 0.46* | *r = -0.09, p = 0.83* |
| 70% | *r = 0.62, p = 0.104* | *r = 0.55, p = 0.157* | *r = 0.19, p = 0.65* | *r = 0.29, p = 0.49* |

**Mean Torque CoV (Single-Joint) - Mean CoViSi (Single-Joint):** *r = 0.12, p = 0.786*

***Torque CoV vs CoViSi (Multi-Joint)***

| **Torque CoV Level** | **10% CoViSi** | **30% CoViSi** | **50% CoViSi** | **70% CoViSi** |
| --- | --- | --- | --- | --- |
| 10% | *r = 0.60, p = 0.114* | *r = -0.43, p = 0.291* | *r = 0.25, p = 0.549* | *r = 0.47, p = 0.239* |
| 30% | *r = 0.24, p = 0.573* | *r = 0.38, p = 0.354* | *r = -0.02, p = 0.968* | *r = 0.58, p = 0.131* |
| 50% | *r = 0.09, p = 0.838* | *r = -0.36, p = 0.381* | *r = 0.34, p = 0.407* | ***r = 0.78, p = 0.022*** |
| 70% | *r = 0.29, p = 0.483* | *r = 0.08, p = 0.843* | *r = 0.24, p = 0.561* | *r = 0.61, p = 0.094* |

**Mean Torque CoV (Multi-Joint) - Mean CoViSi (Multi-Joint):** *r = 0.57, p = 0.144*

***Additional Key Associations***

| **Variables** | **Correlation** | **P-value** |
| --- | --- | --- |
| Mean Pain Intensity (Single-Joint) - Mean Neuromechanical Delay (Single-  Joint) | *r = 0.051* | *p = 0.90* |
| *Kujala Score - Mean Neuromechanical Delay (Single-Joint)* | *r = -0.48* | *p =0.196* |
| *Mean Torque CoV (Single-Joint) - Mean Neuromechanical Delay (Single-*  *Joint)* | *r = 0.051* | *p = 0.90* |
| *Kujala Score - Mean Torque CoV (Single-Joint)* | *r = -0.44* | *p = 0.20* |
| *Kujala Score - Mean Torque CoV (Multi-Joint)* | *r = -0.43* | *p = 0.22* |
| *Kujala Score - CoViSi (Single-Joint)* | ***r = -0.75*** | ***p = 0.032*** |
| *Kujala Score - CoViSi (Multi-Joint)* | *r = -0.712* | *p =0.113* |
| *Mean Pain Intensity (Single-Joint) - Mean XCORR (Single-Joint)* | *r = -0.286* | *p =0.424* |
| *Mean Pain Intensity (Multi-Joint) - Mean XCORR (Multi-Joint)* | *r = 0.06* | *p = 0.879* |
| *Kujala Score - Mean XCORR (Single-Joint)* | *r = 0.57* | *p = 0.08* |
| *Kujala Score - Mean XCORR (Multi-Joint)* | *r = 0.379* | *p =0.315* |
| *Mean Torque CoV (Single-Joint) - Mean XCORR (Single-Joint)* | *r = 0.047* | *p =0.897* |
| *Mean Torque CoV (Multi-Joint) - Mean XCORR (Multi-Joint)* | *r = -0.213* | *p =0.583* |
| *Mean Torque CoV (Pooled) - Mean CoViSi (Pooled)* | *r = 0.24* | *p = 0.56* |
| *Mean CoViSi (Multi-Joint) - Mean XCORR (Multi-Joint)* | *r = 0.17* | *p =0.716* |
| *Mean CoViSi (Single-Joint) - Mean XCORR (Single-Joint)* | *r = -0.132* | *p =0.754* |
