# Supplementary material for: Reduced knee extensor torque steadiness and increased motor unit discharge rate variability in young people with patellofemoral pain: a pilot study
Source: Eur J Appl Physiol. 2025 Dec 22;126(5):2595–615. doi: 10.1007/s00421-025-06083-8 (PMC13236768; doi:10.1007/s00421-025-06083-8)
Supplement: Supplementary file 3 — Supplementary file3 (DOCX 28 KB) [file 421_2025_6083_MOESM3_ESM.docx]

***Number of Motor Units Decomposed***

**Document Overview: This document presents the number of motor units successfully decomposed from vastus medialis (VM) and vastus lateralis (VL) high-density electromyography signals across different torque intensities, exercise types, muscles, and participant groups. Values are shown as mean ± standard deviation, with range (minimum-maximum) reported in parentheses.**

**Data are presented separately for:**

- **Multi-Joint Exercise: Motor unit counts obtained during multi-joint knee extension tasks.**
- **Single-Joint Exercise: Motor unit counts obtained during isolated single-joint knee extension tasks.**
- **Total Motor Units Across Exercises: Motor unit counts summed across both multi-joint and single-joint conditions for each participant.**

**Motor unit counts are reported for both:**

- **VM**
- **VL**

**and for the two participant groups:**

- **Control Group**
- **Patellofemoral Knee Pain (PFP) Group**

*Note: Torque intensities (10%, 30%, 50%, 70%) represent percentage levels of maximum voluntary contraction during testing protocols.*

***Multi-Joint Exercise***

| **Torque Level (%)** | **VM** | **VL** | **VM** | **VL** |
| --- | --- | --- | --- | --- |
|  | **Control Group** | | **PFP Group** | |
| ***10*** | 11.31 ± 3.01  (6-14) | 11.85 ± 6.08  (5-24) | 10.80 ± 5.94  (5-25) | 8.40 ± 8.19  (2-28) |
| ***30*** | 13.54 ± 6.08  (6-22) | 14.46 ± 9.67  (2-36) | 8.90 ± 5.92  (3-19) | 7.44 ± 7.91  (1-21) |
| ***50*** | 12.92 ± 5.84  (4-25) | 15.77 ± 11.91  (2-42) | 12.89 ± 10.29  (2-31) | 9.80 ± 8.35  (1-21) |
| ***70*** | 10.38 ± 7.01  (3-24) | 12.15 ± 9.96  (1-37) | 14.38 ± 9.74  (5-30) | 7.29 ± 6.68  (1-19) |

***Single-Joint Exercise***

| **Torque Level (%)** | **VM** | **VL** | **VM** | **VL** |
| --- | --- | --- | --- | --- |
|  | **Control Group** | | **PFP Group** | |
| ***10*** | 11.38 ± 3.50  (6-19) | 11.77 ± 4.73  (6-21) | 8.80 ± 4.42  (3-16) | 8.80 ± 4.69  (2-19) |
| ***30*** | 11.00 ± 5.55  (3-26) | 13.15 ± 7.44  (2-28) | 11.20 ± 6.25  (4-24) | 5.00 ± 4.17  (2-14) |
| ***50*** | 8.85 ± 4.43  (2-15) | 14.38 ± 8.99  (3-29) | 10.10 ± 7.92  (2-23) | 3.89 ± 3.98  (1-14) |
| ***70*** | 9.08 ± 6.52  (2-20) | 10.54 ± 8.72  (2-33) | 10.63 ± 7.60  (1-20) | 3.63 ± 3.93  (1-13) |

***Total Motor Unit Count Across Exercises***

| **Torque Level (%)** | **VM** | **VL** | **VM** | **VL** |
| --- | --- | --- | --- | --- |
|  | **Control Group** | | **PFP Group** | |
| ***10*** | 22.69 ± 4.63  (18-32) | 23.62 ± 10.19  (15-45) | 19.60 ± 8.13  (10-33) | 17.20 ± 11.07  (4-41) |
| ***30*** | 24.54 ± 9.85  (9-46) | 27.62 ± 15.86  (5-57) | 20.10 ± 11.07  (8-39) | 10.70 ± 11.05  (1-32) |
| ***50*** | 21.77 ± 9.63  (8-39) | 30.15 ± 20.55  (5-69) | 21.70 ± 17.30  (2-47) | 9.33 ± 10.99  (1-30) |
| ***70*** | 18.77 ± 12.85  (3-41) | 22.69 ± 16.86  (3-56) | 25.00 ± 16.16  (6-50) | 10.00 ± 10.13  (1-32) |
